# Orbital Tuning of the β‑Lactam Warhead: Intrinsic n → π* Preactivation in Cephalosporins

**DOI:** 10.1021/acs.jpclett.6c01886

**Published:** 2026-08-17

**Authors:** Sergio Mato, Sofía Municio, José L. Alonso, Elena R. Alonso, Iker León

**Affiliations:** Grupo de Espectroscopía Molecular (GEM), Edificio Quifima, Laboratorios de Espectroscopía y Bioespectroscopia, Facultad de Ciencias, 16782 Universidad de Valladolid, 47011 Valladolid, Spain

## Abstract

The stability–reactivity paradox of β-lactam antibiotics remains a fundamental puzzle in medicinal chemistry: how can cephalosporins maintain high antibacterial efficacy despite having reduced ring strain compared to penicillins? Here, we reveal the intrinsic conformational architecture of 7-aminodesacetoxycephalosporanic acid (7-ADCA), the foundational core of cephalosporins, using laser ablation broadband rotational spectroscopy and high-level quantum chemical calculations. In a striking departure from the hydrogen-bond-mediated activation observed in penicillins, we discovered that 7-ADCA can be electronically preactivated by a subtle n → π* orbital interaction between the carboxylic oxygen and the β-lactam carbonyl. This interaction selectively weakens the amide bond without compromising the scaffold’s overall stability, offering an evolutionary trade-off superior to the fragile penicillin nucleus. A comprehensive survey of the Protein Data Bank (PDB) confirms that this gas-phase geometry not only is the biologically operative pharmacophore but also is adopted by approximately 70% of enzyme-bound cephalosporins. These findings provide insights into the electronic features of the cephalosporin core and offer a structural template consistent with its bioactivity, potentially aiding in the rational design of next-generation antibiotics through subangstrom orbital tuning.

## Graphical abstract

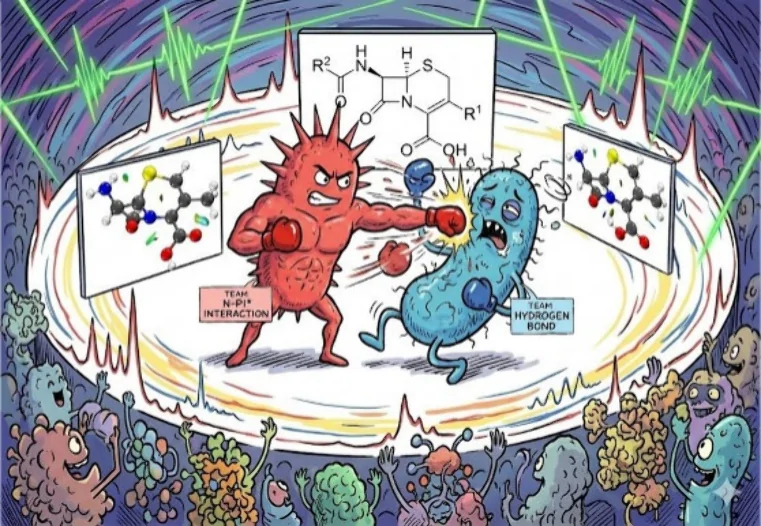

The β-lactam ring is arguably the most significant warhead in the history of medicinal chemistry. For decades, the clinical success of penicillins and cephalosporins has relied on their ability to act as structural mimics of the d-Ala-d-Ala substrate, irreversibly acylating penicillin-binding proteins (PBPs) to halt bacterial cell wall synthesis.^1,2^ However, the rise of antimicrobial resistance (AMR) has turned this chemical race into a delicate balancing act.^1,3–6^ To remain effective, a β-lactam must be reactive enough to acylate its target but stable enough to resist the hydrolytic attack of evolved β-lactamases.^1,2,7^ This “stability–reactivity paradox” is governed by a complex interplay of ring strain, amide resonance, and subtle intramolecular forces that remain difficult to disentangle in condensed phases.

Traditionally, the heightened reactivity of β-lactams has been attributed to the suppression of amide resonance (n_N_ → π*_C=O_) induced by ring strain and the pyramidalization of the bridgehead nitrogen,^2,8,9^ yet this purely geometric view is incomplete. Recent studies have begun to reveal that noncovalent interactions (NCIs), such as hydrogen bonding and n → π* orbital overlaps, act as “electronic rheostats” that can either stabilize the scaffold or preactivate the amide bond for nucleophilic attack.^10,11^ In the case of penicillins, it has been hypothesized that biological activity is intrinsically linked to a specific equatorial conformation,^12–14^ where a trans-annular hydrogen bond effectively “primes” the β-lactam ring for opening. We recently provided experimental evidence supporting this hypothesis.^15^

Cephalosporins, however, present a more intriguing puzzle. Despite having a less-strained six-membered dihydrothiazine ring compared to the five-membered thiazolidine ring of penicillins (see Figure 1), they often exhibit superior pharmacological profiles and enhanced resistance to enzymatic degradation. This suggests that the cephalosporin core (7-ADCA) has evolved a different electronic strategy to manage its reactivity.^13,16^ While synthetic modifications at positions C3 and C7 are known to modulate its spectrum of activity,^2^ the fundamental question remains: what are the intrinsic structural features of the cephalosporin nucleus that underlie its conformational preferences in biological environments?

**Figure 1:**
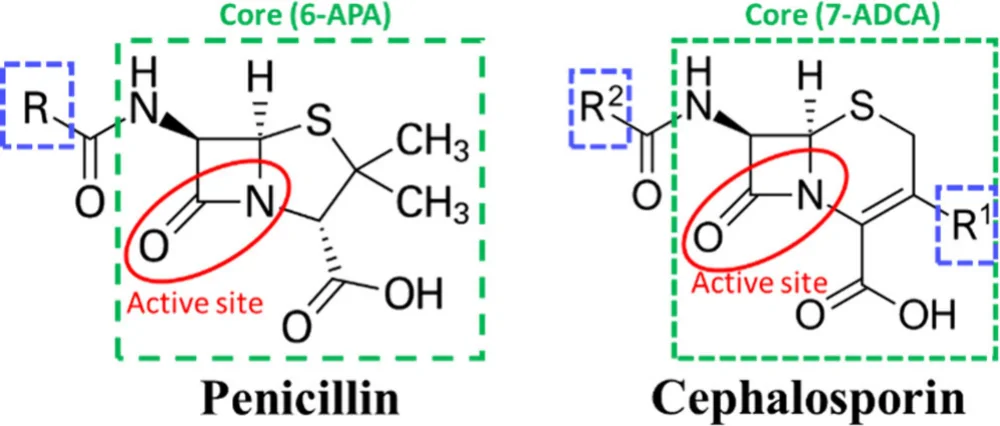
Molecular sketch of penicillin (left) and cephalosporin (right). R accounts for a variable group. The core of 6-APA and 7-ADCA is also indicated, highlighting the carbonyl group, the leading active site of antibiotics.

Herein, we report the first experimental determination of the intrinsic conformational landscape of 7-aminodesacetoxycephalosporanic acid (7-ADCA) using high-resolution broadband rotational spectroscopy in the gas phase. By isolating the molecule from solvent effects and crystal packing forces, we reveal a “molecular tug-of-war” between competing intramolecular forces. In a striking departure from the hydrogen-bond-mediated activation seen in penicillins, we find that the global minimum of 7-ADCA is electronically preactivated by a subtle n → π* interaction between the carboxylic oxygen and the β-lactam carbonyl.

Through a combination of topological analyses (QTAIM, NBO, and NCI) and a comprehensive survey of the Protein Data Bank (PDB), we demonstrate that this gas-phase geometry is not a mere structural curiosity but rather the biologically operative pharmacophore, conserved in approximately 70% of enzyme-bound cephalosporins. Our findings suggest that cephalosporins achieve their therapeutic “sweet spot” through precise orbital tuning, replacing the aggressive activation of penicillins with a more controlled electronic preactivation. This work provides a fundamental structural blueprint for the rational design of next-generation antibiotics, where reactivity is engineered at the subangstrom level.

## Experimental Details

The intrinsic rotational spectrum of 7-ADCA was recorded using a broadband chirped-pulse Fourier transform microwave (LA-CP-FTMW) spectrometer coupled with a laser ablation source.^17–21^ Solid samples of 7-ADCA were pressed into cylindrical pellets and placed in the ablation assembly. The second harmonic of a Nd:YAG laser (355 nm, ∼15 mJ/pulse) was used to vaporize the sample. The resulting plume was immediately entrained in a supersonic expansion of neon (stagnation pressure of 10 bar), which cooled the molecular degrees of freedom to a rotational temperature of approximately 3 K.

The molecular ensemble was polarized by a 4 μs microwave chirp covering the 6–14 GHz frequency range. The free induction decay (FID) was recorded in the time domain for 10 μs and Fourier-transformed to the frequency domain using a Kaiser–Bessel window. A total of 168K FIDs were averaged to achieve a high signal-to-noise ratio. The experimental transitions were fitted to a rigid-rotor Hamiltonian^22^ using Pickett’s SPFIT program.^22^

## Computational Details

The conformational landscape of 7-ADCA was explored through a multistep computational protocol. Initial stochastic searches were performed using molecular mechanics, followed by geometry optimizations and harmonic frequency calculations using Gaussian suite programs.^23^

To ensure a robust description of the electronic structure, calculations were carried out using the density functional theory (DFT) framework with the B3LYP-D3 ^24–26^ and double-hybrid B2PLYPD3 functionals,^27,28^ as well as the ab initio MP2 level of theory,^29^ all employing the 6-311++G­(d,p) basis set.^30^ These methods were selected to provide a balanced treatment of electron correlation and long-range dispersion forces, which are critical for describing noncovalent interactions in medium-sized biomolecules. All stationary points were characterized as true minima by the absence of imaginary frequencies. The experimental rotational parameters were compared with the predicted values to confirm the structural assignments. The Cartesian coordinates and spectroscopic parameters of all of the conformers are listed in Tables S1–S4.

To characterize the intramolecular forces, topological analyses were performed on the optimized wave functions. Quantum theory of atoms in molecules (QTAIM)^31^ and noncovalent interaction (NCI) maps were generated using AIMQB and NCIplot programs,^32,33^ respectively, to identify bond critical points (BCPs) and stabilization regions. Natural bond orbital (NBO) analysis^34^ was employed to quantify the n → π* donor–acceptor interactions and their effect on the β-lactam amide resonance. A PDB survey was conducted by extracting all cephalosporin–ligand complexes with a resolution better than 2.5 Å, analyzing the O=C–C–C dihedral angle, and calculating the heavy-atom RMSD against the gas-phase structures.

*Deciphering the Conformational Landscape of 7-ADCA*. To unveil the intrinsic structural preferences of the cephalosporin core, we explored the potential energy surface (PES) of 7-ADCA. The landscape is characterized by a delicate competition between the orientation of the exocyclic amino group and the carboxylic moiety (Figure 2). Our computational survey identified one primary structural motif depending on the *cis*/*trans* position of the carboxyl group. The *trans* carboxylic conformers are stabilized by a classical O–H···O=C_lactam_ interaction, while the *cis* carboxylic conformers are stabilized by the hydrogen bond with the carbonyl group of the same acidic functional group (*cis*-carboxylic), seemingly lacking the interaction with the lactam ring. Additionally, within those configurations, the amino group can point toward the O=C_lactam_ functional group and away from the sulfur atom (type a) or vice versa (type b).

**Figure 2:**
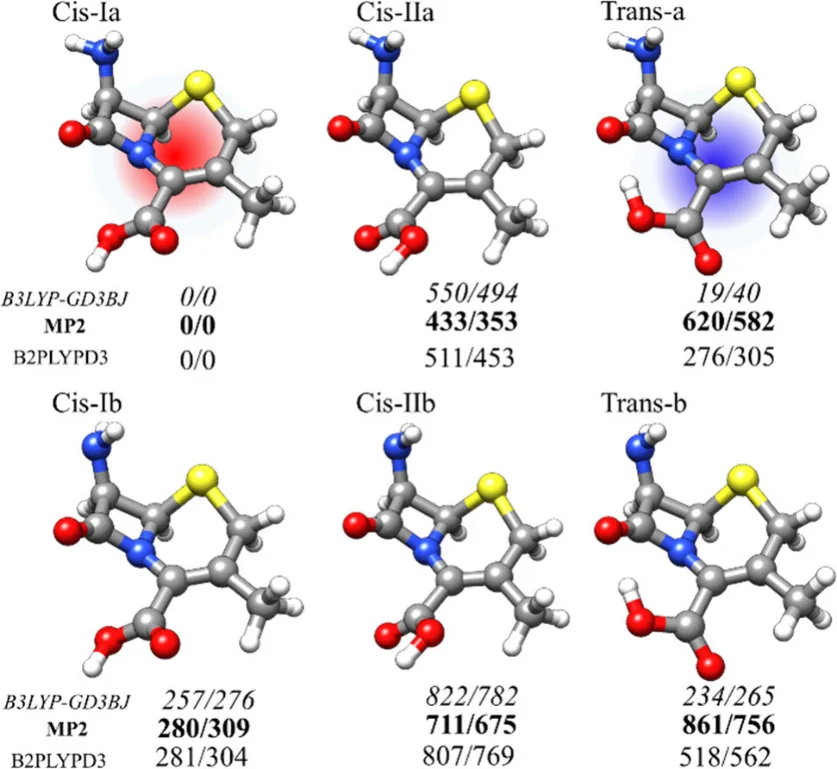
Conformational panorama of 7-ADCA and their relative energies (Δ*E*_ZPE_/Δ*G*) in cm^–1^ using B3LYP, MP2, and B2PLYPD methods. The detected species are highlighted with colored backgrounds.

While conventional chemical intuition often prioritizes hydrogen-bonded species, our laser ablation broadband rotational spectrum (LA-CP-FTMW) shown in Figure 3 and Figure S1 revealed a more complex reality. Analysis of the rotational transitions in Table 1 allowed for the unambiguous identification of two distinct rotamers in the gas phase. The experimental rotational constants (*A*, *B*, and *C*) and the ^14^N nuclear quadrupole coupling tensors (χ_ii_),^35^ which serve as a high-sensitivity probe of the local electronic environment, provided an excellent match with the predicted parameters (see Table 1) for the two most stable species, Cis-Ia (rotamer I) and Trans-a (rotamer II). Remarkably, the Cis-Ia species, the non-H-bonded form of the lactam carbonyl group, emerges as the global minimum, suggesting that the structural integrity of the cephalosporin scaffold is governed by subtle electronic effects that outweigh traditional electrostatic stabilization.

**Figure 3:**
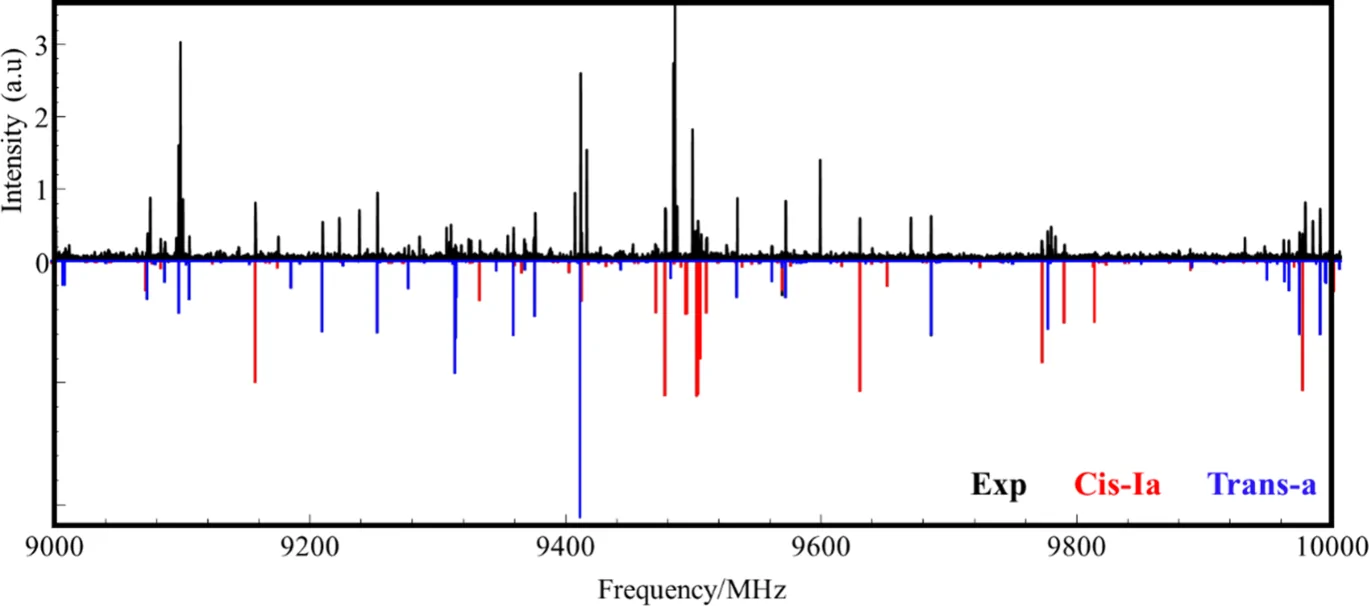
Section of rotational spectra of 7-ADCA in the 9–10 GHz range together with the predicted spectrum of Cis-Ia (red) and Trans-a (blue) conformers. The whole spectrum is shown in Figure S1.

**Table 1:** Experimental and Calculated (MP2/6-311++G­(d,p)) Spectroscopic Parameters for the Detected Conformer of 7-ADCA

	experimental		calculated	
	rotamer I	rotamer II	Cis-Ia	Trans-a
*A* ^a^	887.3841349(188)	896.81579(52)^f^	887	904
*B*	591.6751667(103)	586.524432(270)	591	581
*C*	387.9536932(111)	378.450446(201)	390	381
μ_a_ ^b^	not observed	observed	0.4	3.8
μ_b_	observed	observed	1.5	2.8
μ_c_	observed	not observed	2.2	0.6
χ_aa(r)_ ^c^	1.9793(28)	1.726(120)	1.99	1.83
χ_bb(r)_	0.6960(25)	1.033(96)	0.72	0.80
χ_cc(r)_	–2.6753(25)	–2.759(96)	–2.71	–2.63
χ_aa(a)_	0.2289(33)	0.731(97)	0.47	0.90
χ_bb(a)_	–1.5874(27)	–1.965(92)	–1.69	–2.04
χ_cc(a)_	1.3585(27)	1.235(92)	1.22	1.14
*N* ^d^	124	151		
σ^e^	31.3	27.2		

a *A*, *B*, and *C* represent the rotational constants (in megahertz).

b μ_a_, μ_b_, and μ_c_ are the electric dipole moment components in debyes (observed or not observed for experimental values).

c χ_aa_, χ_bb_, and χ_cc_, are the diagonal elements of the ^14^N nuclear quadrupole coupling tensor (in megahertz). (r) and (a) correspond to the β-lactam ring and amine ^14^N nuclei, respectively.

d RMS deviation of the fit (in kilohertz).

e Number of measured transitions.

f Standard error in parentheses expressed in units of the last digit.

No more conformers were observed, in good agreement with their higher relative energy at the B3LYP-D3/6-311++G­(d,p) level, which suggests that their population is negligible. In fact, a comparative analysis of the different levels of theory reveals a notable dichotomy between structural precision and energetic prediction. While the MP2 method provides the most accurate rotational constants, showing an excellent agreement with the experimental values (Table 1), it appears to overestimate the energy gap between the two detected species, placing the Trans-a conformer approximately 620 cm^–1^ above the global minimum. In contrast, the B3LYP functional predicts the two species to be nearly isoenergetic (Δ*E* ≈ 19 cm^–1^), which is more consistent with the simultaneous experimental observation of both rotamers with significant signal intensity in the supersonic expansion and the estimate that the Trans-a conformer is ≈100 cm^–1^ above Cis-Ia. The double-hybrid B2PLYP functional offers a balanced middle ground, yielding high-quality spectroscopic parameters and an intermediate energy difference (Δ*E* ≈ 492 cm^–1^). These discrepancies highlight the extreme sensitivity of the 7-ADCA potential energy surface to the treatment of electron correlation and dispersion forces, justifying our multimethod approach to ensure a reliable conformational assignment.

We note that amine inversion between Cis-Ib and Cis-Ia is only ∼350 cm^–1^ (see Figure S2), explaining its nondetection. Tables S5 and S6 collect all of the measured frequencies.

n → π* Interaction: An Electronic Preactivation Mechanism. To rationalize why both configurations are thermodynamically preferred and how this relates to its antibiotic function, we conducted a suite of topological analyses (NCI, QTAIM, and NBO). The results are shown in Figure 4 and Tables S7–S9. A sharp dichotomy in the stabilization motifs of the two detected species became evident. In the Trans-a conformer, the β-lactam ring is “locked” by the strong O–H···O=C hydrogen bond. While this interaction provides overall molecular stability, QTAIM analysis reveals that it reinforces the N_1_–C_8_ amide bond with an electron density at BCP of 0.299 au, effectively deactivating the chemical warhead.

**Figure 4:**
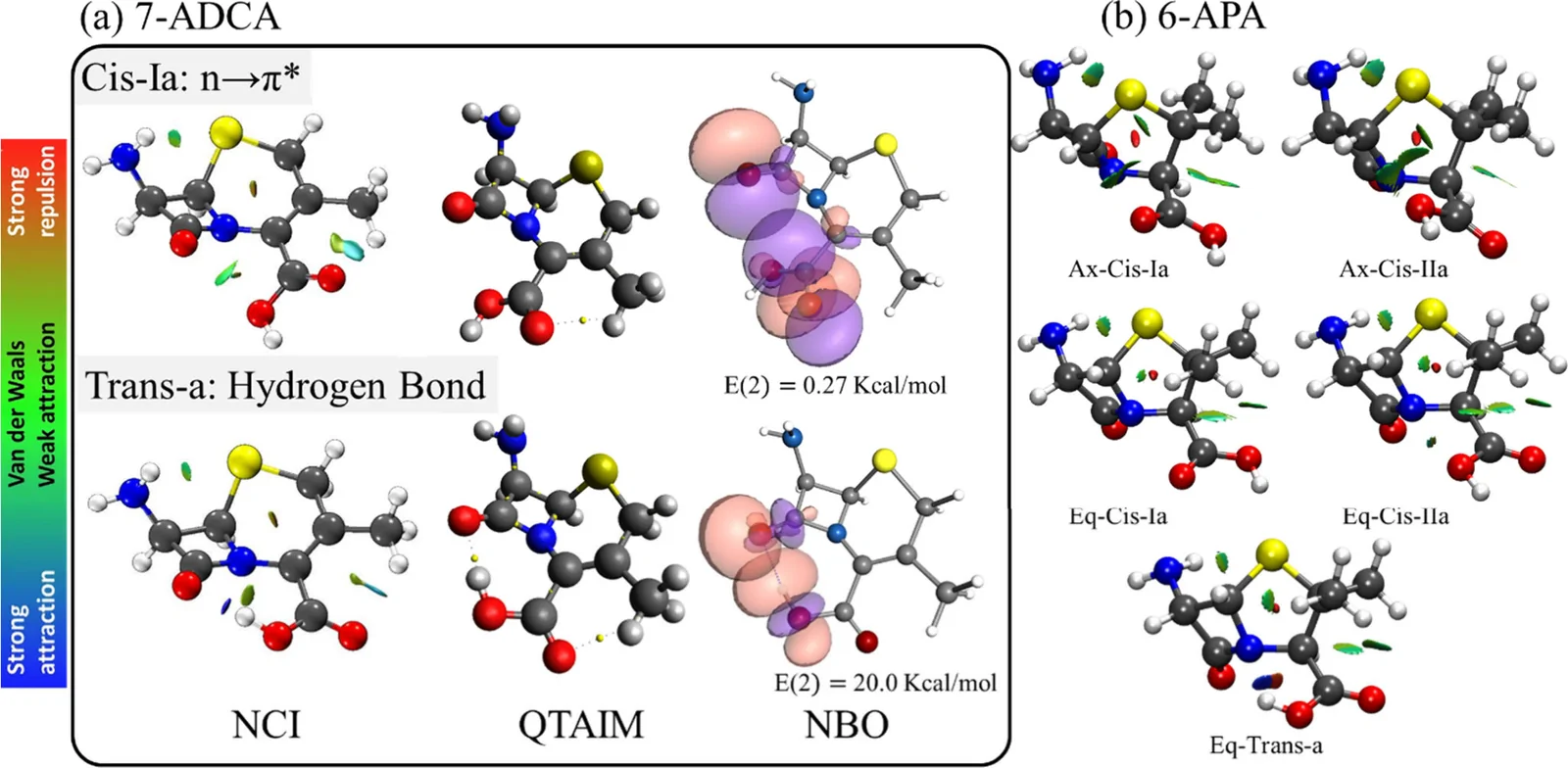
(a) Intramolecular interaction analysis of the detected species of 7-ADCA by NCI, QTAIM, and NBO. (b) NCI plot analysis of the most stable conformers of 7-ADCA.

In stark contrast, the Cis-Ia global minimum achieves stabilization through a specific n → π* orbital interaction between the carboxylic oxygen lone pair (n_O_) and the β-lactam carbonyl antibonding orbital (π*_C=O_), analogous to that previously reported for aspirin.^36^ NBO analysis confirms that this trans-annular overlap induces a depletion of electron density within the β-lactam ring. Crucially, the amide bond in the *cis* form is intrinsically weaker (ρ = 0.289 au) and more single-bond-like than in the *trans* form. This identifies the n → π* interaction as an electronic preactivation mechanism. The molecule is “primed” for the nucleophilic attack required to acylate its enzymatic target even in its isolated, ground state geometry.

To provide a rigorous quantitative benchmark for the reactivity of the 7-ADCA core, we analyzed the structural and topological properties of the β-lactam C–N bond (Table 2). This specific bond constitutes the critical active site of the warhead, where cleavage occurs to form the covalent bond with penicillin-binding proteins (PBPs).^14^ As shown in Table 2, standard amides (acetamide) and the Ala-Ala dipeptide,^37^ the natural d-Ala-d-Ala substrate mimic,^1,2^ exhibit shorter and more robust C–N bonds (≈1.35–1.37 Å; ρ ≈ 0.31–0.33 au).

**Table 2:** Structural and Topological Parameters (MP2/6-311++G­(d,p)) for the Amide Bond in 7-ADCA, 6-APA, and Model Systems

	bond distance (Å)		ρ (au)		net charge (*e*)		
molecule	C–N	C=O	C–N	C=O	O	C	N
acetamide^a^	1.378	1.221	0.3062	0.3982	–1.149	1.459	–1.193
β-lactam^a^	1.378	1.208	0.3037	0.4058	–1.159	1.476	–1.159
Ala-Ala (Trans-I)^b^	1.347	1.240	0.3303	0.3909	–1.168	1.373	–1.150
Ala-Ala (Cis-I)^b^	1.357	1.230	0.3160	0.3894	–1.166	1.442	–1.254
6-APA (Ax-Cis-Ia)^c^	1.409	1.208	0.2920	0.4040	–1.139	1.373	–1.065
6-APA (Eq-trans-a)^c^	1.431	1.202	0.2840	0.4090	–1.117	1.353	–1.056
7-ADCA (Cis-Ia)^d^	1.408	1.205	0.2900	0.4060	–1.128	1.394	–1.142
7-ADCA (Trans-a)^d^	1.391	1.217	0.3000	0.3960	–1.141	1.379	–1.165

a Calculated in this work.

b From ref 34.

c From ref 14.

d This work.

In contrast, the β-lactam ring in 7-ADCA (Cis-Ia) shows a significant bond elongation (1.408 Å) and a decrease in electron density at the BCP (ρ = 0.2900 au). This structural “weakening” is directly induced by the trans-annular n → π* interaction, which is absent in the Trans-a conformer (1.391 Å; ρ = 0.3000 au). While this electronic activation is less aggressive than the extreme fragility observed in the 6-APA penicillin core (ρ = 0.2840 au for its equatorial form^15^), it confirms that cephalosporins achieve a unique “sweet spot”. They are electronically preactivated enough to lethally acylate their targets yet sufficiently stable to avoid the rapid degradation that characterizes more strained or H-bond-activated β-lactams.

We highlight the fact that these extrapolations from the gas phase to the condensed phase can be done due to the remarkable similarity observed between the Cis-Ia structure and that observed in enzymes, as shown in Figure 5a, where a comparison between the Cis-Ia structure superimposed with the 2ZQ9 enzyme is done.

**Figure 5:**
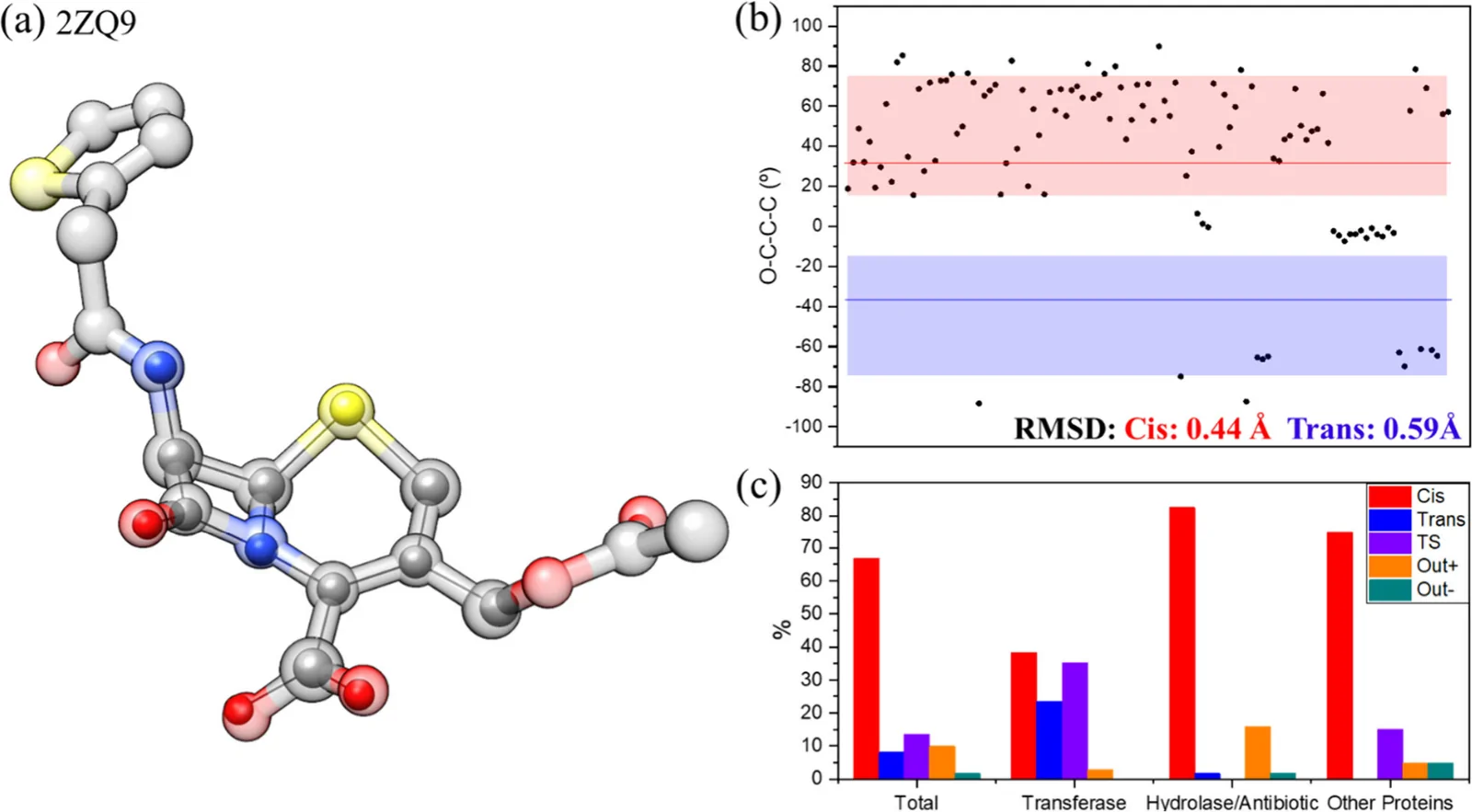
(a) Comparison of a crystallized cephalosporin compound in the 2ZQ9 enzyme (transparent) and the Cis-Ia gas-phase structure (solid spheres). (b) Scattering plot of O–C–C–C dihedral angle in collected PDB cephalosporin compounds. The red and blue lines correspond with the dihedral value in the Cis-Ia and Trans-a conformations, respectively. The colored background is the interval of classification for each structure. (c) Relative abundance of classification structures in the enzymes.

*Conformational Conservation and Comparison with Enzyme-Bound Structures*. It is important to note that the desolvated gas-phase environment serves as an excellent model for the enzyme active site. It has been proven that the shielded microenvironment of a protein pocket exhibits an exceptionally low effective dielectric constant and highly restricted electrostatic screening due to local desolvation and active-site shielding.^38–40^ Thus, the intrinsic electronic signatures identified here are more representative of the molecule’s behavior at the drug–target interface than its behavior in bulk aqueous solution.

The quantitative analysis presented in Table 2 provides definitive evidence for this preactivation. The systematic elongation of the C–N bond and the depletion of electron density (ρ) in the Cis-Ia conformer, compared to both the Trans-a form and standard amides, demonstrate that the n → π* interaction has a direct, measurable impact on the chemical warhead. This structural “priming” explains how cephalosporins maintain high efficacy. The core is preorganized into its active state even before reaching the enzymatic target.

To validate the pharmacological relevance of our gas-phase findings, we interrogated the geometries of cephalosporins within protein active sites using the PDB. A survey of more than 111 cephalosporin–ligand complexes (see Figure S3) reveals a striking structural conservation. Approximately 70% of enzyme-bound cephalosporins adopt a conformation analogous to our gas-phase Cis-Ia minimum, regardless of the specific protein environment or the nature of the substituents at C3 and C7 (Figure 5b,c). In contrast, only approximately 5% of the cephalosporins adopt a conformation analogous to the Trans-a minimum geometry even when allowing a large interval in the angle that classifies this configuration (see Figure 5b).

The structural similarity is quantitatively reinforced by RMSD analysis, which shows that the biologically operative state exhibits a significantly higher fidelity to the intrinsic Cis-Ia structure (averaged RMSD of 0.44 Å) than to the H-bonded *trans* form (averaged RMSD of 0.59 Å). Because the difference in the value considers all atoms and therefore is mainly due to the relative position of the acidic group due to the interactions with the β-lactam, a difference of 0.15 Å is remarkable. This suggests that the solvent-free Cis-Ia geometry reflects a preorganized state that is highly conserved across different protein environments. The cephalosporin core appears to be “preorganized” for binding. The molecule’s inherent electronic structure is consistent with a preactivated state prior to enzymatic binding.

The structural fidelity is further reinforced by the analysis of n → π* structural hallmarks. A survey of 111 PDB complexes (see Table S10) yields an average *d*_O···C_ distance of 3.36 Å and a Bürgi–Dunitz-like angle (α_O···C=O_) of 118°. These values show a remarkable agreement with the intrinsic parameters of the Cis-Ia conformer (3.25 Å and 123°, respectively). In contrast, the Trans-a form exhibits an angle of 168°, which is geometrically inconsistent with the distribution observed in enzyme-bound cephalosporins. This confirms that the n → π* interaction is not a gas-phase curiosity but the fundamental force that preorganizes the core into its biologically operative state.

*Environmental Effects and Solvent Robustness*. While the primary goal of this study is to isolate the intrinsic properties of 7-ADCA, we assessed the influence of the environment using implicit solvation calculations (IEFPCM model for water^41^). The results (see Table S11) show that the Cis-Ia motif remains more stable than the *trans* structures even in a polar environment, confirming that the n → π* preactivation is an inherent and robust feature of the cephalosporin core.

Another observation is that there is a notable stabilization of the b-type structures over the a-type ones in a polar environment, and Cis-Ib becomes isoenergetic with Cis-Ia. Trans-Ib is also stabilized and becomes more stable than Trans-Ia. Thus, it is interesting to observe how condensed phases stabilize those structures (*cis* and *trans*) with the amino group interacting with the sulfur atom over those structures interacting with the carbonyl group.

To go beyond continuum models, we explored microsolvated 7-ADCA clusters with up to three explicit water molecules (Tables S12–S14 and Figure S4). These calculations demonstrate that the n_O_ → π_C=O_* interaction is not suppressed upon hydration but remains a persistent electronic feature. The *cis* arrangement is preferentially stabilized in all hydrated clusters, with the energy gap relative to *trans* species increasing to approximately 1000 cm^–1^. The strength of the interaction is modulated by the hydrogen-bonding topology. Hydration motifs that engage the carboxylic lone pairs can slightly weaken the n → π* overlap, while alternative patterns preserve it. Crucially, hydration does not erase the intrinsic orbital preactivation but confirms its robustness in complex environments.

*Cephalosporin Advantage: Tuning the Stability–Reactivity Paradox*. A direct comparison between the 7-ADCA nucleus and the previously characterized penicillin nucleus (6-APA) provides a molecular rationale for the superior therapeutic profile of cephalosporins.

- Conformational Rigidity. While penicillins feature a flexible five-membered thiazolidine ring and five conformers were observed, the six-membered dihydrothiazine ring of cephalosporins locks the scaffold into two conformers where the most stable structure is the biologically active n → π* geometry, minimizing the entropic penalty upon target binding.
- Orbital Tuning versus Ring Strain. Penicillins achieve high reactivity through an “aggressive” activation mechanism involving strong hydrogen bonds and extreme ring strain, which renders the ring fragile and susceptible to β-lactamase-mediated hydrolysis (ρ = 0.284 au).
- The “Sweet Spot”. Cephalosporins, by replacing aggressive H-bond activation with subtle n → π* orbital tuning, achieve an intermediate bond strength (ρ = 0.289 au).

This electronic reality identifies the cephalosporin core as an evolutionary “sweet spot”. It is robust enough to resist enzymatic degradation yet sufficiently preactivated to lethally acylate penicillin-binding proteins. This transition from strain-induced instability to orbital-tuned reactivity represents a fundamental principle in the structural evolution of β-lactam antibiotics.

The identification of this orbital-based activation blueprint opens new avenues for the rational design of next-generation antibiotics. We propose that by introducing specific substituents at position C3 or C7, the electron density of the donor oxygen or the electrophilicity of the β-lactam carbonyl could be precisely tuned. For instance, incorporating electron-donating groups that enhance the n → π* overlap could “prime” the ring for faster acylation without the need for increased ring strain, which often leads to chemical instability. This subangstrom orbital tuning offers a pathway to design antibiotics that are both more resistant to β-lactamases and more lethal to their targets.

In conclusion, we have provided the first experimental evidence of the intrinsic conformational landscape of the cephalosporin core (7-ADCA). By isolating the molecule in the gas phase, we have identified a “molecular tug-of-war” between stabilizing hydrogen bonds and activating orbital interactions, where the latter dictates the global minimum and the biological operative structure.

Our results offer a molecular-level explanation for the “stability–reactivity paradox” that defines the clinical superiority of cephalosporins. While penicillins achieve high reactivity through a fragile, highly strained nucleus, cephalosporins could utilize a more robust six-membered scaffold that is electronically “tuned” via n → π* interactions. This subtle activation provides a perfect balance. The ring is stable enough to resist the hydrolytic action of β-lactamases yet sufficiently preactivated to lethally acylate its bacterial targets. This work demonstrates that the efficacy of β-lactam antibiotics appears to be significantly influenced by subangstrom electronic effects and specific orbital tuning.

The identification of this orbital-based activation blueprint opens new avenues for the rational design of next-generation antibiotics, where reactivity can be precisely modulated by targeting specific noncovalent interactions within the core scaffold.

## Supplementary Material



## Notes

Measured frequencies and residuals for the rotational transitions of detected conformers and species (PDF)

## Abbreviations

7-ADCA: 7-aminodesacetoxycephalosporanic acid
PDB: Protein Data Bank
mp: melting point
LA-CP-FTMW: laser ablation chirped-pulse Fourier transform microwave
AWG: arbitrary waveform generator
SSD: solid state amplifier
FIDs: free induction decays
DFT: density functional theory
PES: potential energy surface
ZPE: zero-point energy
NBO: natural bond orbital
QTAIM: quantum theory of atoms in molecules
NCI: noncovalent interaction
