# Supplementary material for: Orbital Tuning of the β‑Lactam Warhead: Intrinsic n → π* Preactivation in Cephalosporins
Source: J Phys Chem Lett. 2026 Aug 17;17(34):9919–26. doi: 10.1021/acs.jpclett.6c01886 (PMC13528287; doi:10.1021/acs.jpclett.6c01886)

Supporting Information for:

Orbital Tuning of the β-Lactam Warhead: Intrinsic n→π* Pre-activation in Cephalosporins.

Sergio Mato,[a] Sofía Municio,[a] José Luis Alonso,[a] Elena R. Alonso,[a] Iker León,[a]*

[a] *Grupo de Espectrocopía Molecular (GEM), Edificio Quifima, Laboratorios de Espectroscopia y Bioespectroscopia, Parque Científico UVa, Universidad de Valladolid, 47011, Valladolid, Spain.*

Table S1. Theoretical spectroscopic parameters of 7-ADCA at B3PLYP-D3(BJ)/6-311++(d,p).

Parameters	Cis-Ia	Trans-a	Trans-b	Cis-Ib	Cis-IIa	Cis-IIb
A^a	878.0	886.7	884.9	875.3	883.3	880.2
B	588.9	584.5	584.7	589.6	579.9	580.6
C	383.8	375.7	375.8	384.0	384.2	384.5
$\|\mu_a\|^b$	0.5	4.0	4.3	0.8	0.9	0.5
$\|\mu_b\|$	1.9	3.1	5.0	3.7	3.3	5.1
$\|\mu_c\|$	2.4	0.6	1.3	3.1	0.6	1.3
$\chi_{aa(r)}{}^c$	2.06	1.86	1.88	2.08	2.09	2.11
$\chi_{bb(r)}$	0.81	0.97	0.93	0.78	0.88	0.85
$\chi_{cc(r)}$	-2.88	-2.83	-2.81	-2.86	-2.97	-2.95
$\chi_{aa(a)}$	0.40	0.70	2.67	2.61	0.35	2.62
$\chi_{bb(a)}$	-1.58	-1.92	-4.75	-4.61	-1.66	-4.66
$\chi_{cc(a)}$	1.18	1.22	2.08	2.00	1.31	2.05
ΔE^d	27	0	244	316	583	889
ΔE_{ZPE}	0	19	234	257	550	822
ΔG	0	40	265	276	494	782

[a]A, B, and C represent the rotational constants (in MHz); [b]μ_a, μ_b and μ_c are the electric dipole moment components in Debyes (observed or not observed for experimental values). [c]χ_{aa}, χ_{bb}, and χ_{cc}, are the diagonal elements of the ^{14}N nuclear quadrupole coupling tensor (in MHz); (r) and (a) correspond to the β-lactam ring and amine ^{14}N nuclei, respectively. Standard error in parentheses expressed in units of the last digit. [d]ΔE, ΔE_{ZPE}, ΔG are the electronic energy difference, the energy difference including zero-point vibrational corrections, and the Gibbs free energy difference (1 atm, 298 K), respectively.

Table S2. Theoretical spectroscopic parameters of 7-ADCA at MP2/6-311++(d,p).

Parameters	Cis-Ia	Cis-IIb	Cis-IIa	Trans-a	Cis-IIb	Trans-b
A^a	887.0	883.9	891.9	903.6	888.2	901.4
B	590.8	591.6	584.0	581.4	584.8	582.0
C	389.6	389.8	390.1	380.8	390.4	381.0
$\|\mu_a\|^b$	0.4	0.7	0.7	3.8	0.4	4.0
$\|\mu_b\|$	1.5	3.5	2.7	2.8	4.7	4.9
$\|\mu_c\|$	2.2	3.0	0.6	0.6	1.3	1.3
$\chi_{aa(r)}{}^c$	1.99	2.01	2.01	1.83	2.03	1.86
$\chi_{bb(r)}$	0.72	0.70	0.78	0.80	0.76	0.78
$\chi_{cc(r)}$	-2.71	-2.70	-2.79	-2.63	-2.79	-2.64
$\chi_{aa(a)}$	0.47	2.35	0.43	0.90	2.35	2.41
$\chi_{bb(a)}$	-1.69	-4.28	-1.78	-2.04	-4.32	-4.36
$\chi_{cc(a)}$	1.22	1.93	1.35	1.14	1.97	1.95
ΔE^d	0	293	433	554	745	814
ΔE_{ZPE}	0	280	415	620	711	861
ΔG	0	309	353	582	675	756

[a]A, B, and C represent the rotational constants (in MHz); [b]μ_a, μ_b and μ_c are the electric dipole moment components in Debyes (observed or not observed for experimental values). [c]χ_{aa}, χ_{bb}, and χ_{cc}, are the diagonal elements of the ^{14}N nuclear quadrupole coupling tensor (in MHz); (r) and (a) correspond to the β-lactam ring and amine ^{14}N nuclei, respectively. Standard error in parentheses expressed in units of the last digit. [d]ΔE, ΔE_{ZPE}, ΔG are the electronic energy difference, the energy difference including zero-point vibrational corrections, and the Gibbs free energy difference (1 atm, 298 K), respectively.

Table S3. Theoretical spectroscopic parameters of 7-ADCA at B2PLYPD3/6-311++(d,p).

Parameters	Cis-Ia	Cis-Ib	Trans-a	Cis-IIa	Trans-b	Cis-IIb
A^a	881.1	878.2	892.6	886.5	890.3	883.0
B	589.4	590.2	583.2	581.0	583.6	581.9
C	385.6	385.8	377.5	386.0	377.6	386.4
$\|\mu_a\|^b$	0.5	0.8	4.0	0.8	4.2	0.5
$\|\mu_b\|$	1.7	3.7	3.0	3.1	5.0	5.0
$\|\mu_c\|$	2.3	3.1	0.6	0.6	1.3	1.3
$\chi_{aa(r)}^c$	2.04	2.06	1.86	2.07	1.88	2.09
$\chi_{bb(r)}$	0.79	0.76	0.92	0.85	0.89	0.82
$\chi_{cc(r)}$	-2.83	-2.82	-2.78	-2.92	-2.77	-2.92
$\chi_{aa(a)}$	0.46	2.52	0.78	0.42	2.58	2.53
$\chi_{bb(a)}$	-1.68	-4.50	-1.98	-1.77	-4.62	-4.55
$\chi_{cc(a)}$	1.22	1.98	1.20	1.34	2.04	2.02
ΔE^d	0	346	434	579	733	941
ΔE_{ZPE}	0	320	492	569	768	904
ΔG	0	343	520	511	812	865

[a]A, B, and C represent the rotational constants (in MHz); [b]μ_a, μ_b and μ_c are the electric dipole moment components in Debyes (observed or not observed for experimental values). [c]χ_{aa}, χ_{bb}, and χ_{cc}, are the diagonal elements of the ^{14}N nuclear quadrupole coupling tensor (in MHz); (r) and (a) correspond to the β-lactam ring and amine ^{14}N nuclei, respectively. Standard error in parentheses expressed in units of the last digit. [d]ΔE, ΔE_{ZPE}, ΔG are the electronic energy difference, the energy difference including zero-point vibrational corrections, and the Gibbs free energy difference (1 atm, 298 K), respectively.

Table S4. Cartesian coordinates in Angstroms (Å) of the most stable conformers of 7-ADCA at MP2/6-311++G (d,p) level of theory.

Cis-Ia				Cis-IIac				Trans-a			
Atom	X	Y	Z	Atom	X	Y	Z	Atom	X	Y	Z
C1	-1.042	-0.006	0.167	C	-1.024	-0.020	0.211	C	-1.072	-0.044	0.143
C2	-1.278	1.324	0.024	C	-1.269	1.304	0.044	C	-1.311	1.269	-0.110
C3	-0.175	2.350	0.142	C	-0.166	2.336	0.119	C	-0.238	2.330	-0.016
S4	1.506	1.775	-0.243	S	1.509	1.758	-0.287	S	1.479	1.793	-0.277
C5	1.383	0.339	0.854	C	1.412	0.350	0.849	C	1.339	0.416	0.883
N6	0.229	-0.490	0.512	N	0.254	-0.488	0.550	N	0.230	-0.471	0.509
C7	1.007	-1.467	-0.139	C	1.021	-1.478	-0.097	C	1.073	-1.431	-0.040
C8	2.306	-0.850	0.442	C	2.329	-0.848	0.447	C	2.318	-0.759	0.571
O9	0.723	-2.353	-0.903	O	0.722	-2.375	-0.842	O	0.851	-2.378	-0.771
C10	-2.638	1.918	-0.227	C	-2.635	1.900	-0.173	C	-2.669	1.828	-0.438
N11	3.429	-0.569	-0.413	N	3.437	-0.587	-0.433	N	3.476	-0.479	-0.233
C12	-2.102	-1.046	0.023	C	-2.035	-1.120	0.163	C	-2.153	-1.104	0.142
O13	-3.065	-0.966	-0.710	O	-2.017	-2.080	0.900	O	-3.268	-0.874	0.553
O14	-1.862	-2.101	0.834	O	-2.961	-0.937	-0.809	O	-1.821	-2.318	-0.318
H15	1.399	0.653	1.902	H	1.449	0.690	1.887	H	1.279	0.772	1.915
H16	-0.382	3.154	-0.574	H	-0.389	3.125	-0.609	H	-0.426	3.085	-0.786
H17	-0.189	2.803	1.142	H	-0.161	2.809	1.109	H	-0.314	2.839	0.954
H18	2.643	-1.393	1.330	H	2.680	-1.371	1.342	H	2.620	-1.257	1.497
H19	-2.747	2.835	0.363	H	-2.756	2.195	-1.223	H	-3.417	1.055	-0.602
H20	-2.738	2.190	-1.284	H	-2.733	2.806	0.437	H	-3.019	2.463	0.385
H21	-3.450	1.234	0.017	H	-3.443	1.217	0.082	H	-2.593	2.460	-1.330
H22	4.045	-1.373	-0.470	H	4.055	-1.390	-0.478	H	4.123	-1.260	-0.219
H23	3.126	-0.343	-1.355	H	3.118	-0.388	-1.376	H	3.225	-0.295	-1.199
H24	-2.545	-2.755	0.624	H	-3.551	-1.704	-0.753	H	-0.893	-2.362	-0.633

Cis-Ib				Cis-IIb				Trans-b			
Atom	X	Y	Z	Atom	X	Y	Z	Atom	X	Y	Z
C	-1.040	-0.005	0.169	C	-1.022	-0.017	0.213	C	-1.070	-0.044	0.144
C	-1.276	1.326	0.028	C	-1.264	1.309	0.046	C	-1.310	1.269	-0.110
C	-0.175	2.355	0.139	C	-0.162	2.341	0.116	C	-0.241	2.335	-0.022
S	1.503	1.771	-0.249	S	1.510	1.752	-0.290	S	1.474	1.791	-0.280
C	1.379	0.341	0.854	C	1.409	0.348	0.849	C	1.334	0.420	0.884
N	0.231	-0.489	0.513	N	0.255	-0.488	0.550	N	0.232	-0.470	0.511
C	1.010	-1.463	-0.147	C	1.020	-1.477	-0.105	C	1.078	-1.427	-0.048
C	2.309	-0.862	0.441	C	2.330	-0.865	0.445	C	2.322	-0.768	0.570
O	0.718	-2.347	-0.909	O	0.711	-2.372	-0.845	O	0.850	-2.369	-0.778
C	-2.637	1.919	-0.220	C	-2.629	1.906	-0.170	C	-2.672	1.823	-0.433
N	3.409	-0.747	-0.468	N	3.414	-0.771	-0.486	N	3.463	-0.658	-0.286
C	-2.102	-1.044	0.025	C	-2.038	-1.114	0.166	C	-2.151	-1.103	0.145
O	-3.063	-0.963	-0.709	O	-2.030	-2.065	0.913	O	-3.265	-0.871	0.557
O	-1.863	-2.098	0.838	O	-2.953	-0.935	-0.817	O	-1.820	-2.316	-0.315
H	1.392	0.659	1.901	H	1.444	0.691	1.887	H	1.271	0.778	1.915
H	-0.383	3.154	-0.582	H	-0.384	3.126	-0.616	H	-0.430	3.083	-0.798
H	-0.184	2.813	1.136	H	-0.152	2.819	1.104	H	-0.315	2.851	0.944

H	2.623	-1.406	1.338	H	2.660	-1.390	1.347	H	2.603	-1.268	1.502
H	-2.745	2.837	0.369	H	-2.750	2.205	-1.218	H	-3.409	1.047	-0.628
H	-2.741	2.189	-1.278	H	-2.730	2.808	0.444	H	-3.040	2.427	0.406
H	-3.448	1.235	0.029	H	-3.438	1.221	0.082	H	-2.596	2.483	-1.305
H	3.152	-0.257	-1.318	H	3.142	-0.297	-1.341	H	3.248	-0.183	-1.156
H	4.210	-0.291	-0.046	H	4.223	-0.308	-0.088	H	4.240	-0.194	0.169
H	-2.545	-2.753	0.627	H	-3.545	-1.701	-0.762	H	-0.892	-2.360	-0.630

Figure S1. Rotational spectra of 7-ADCA in the 6 - 14 GHz range.

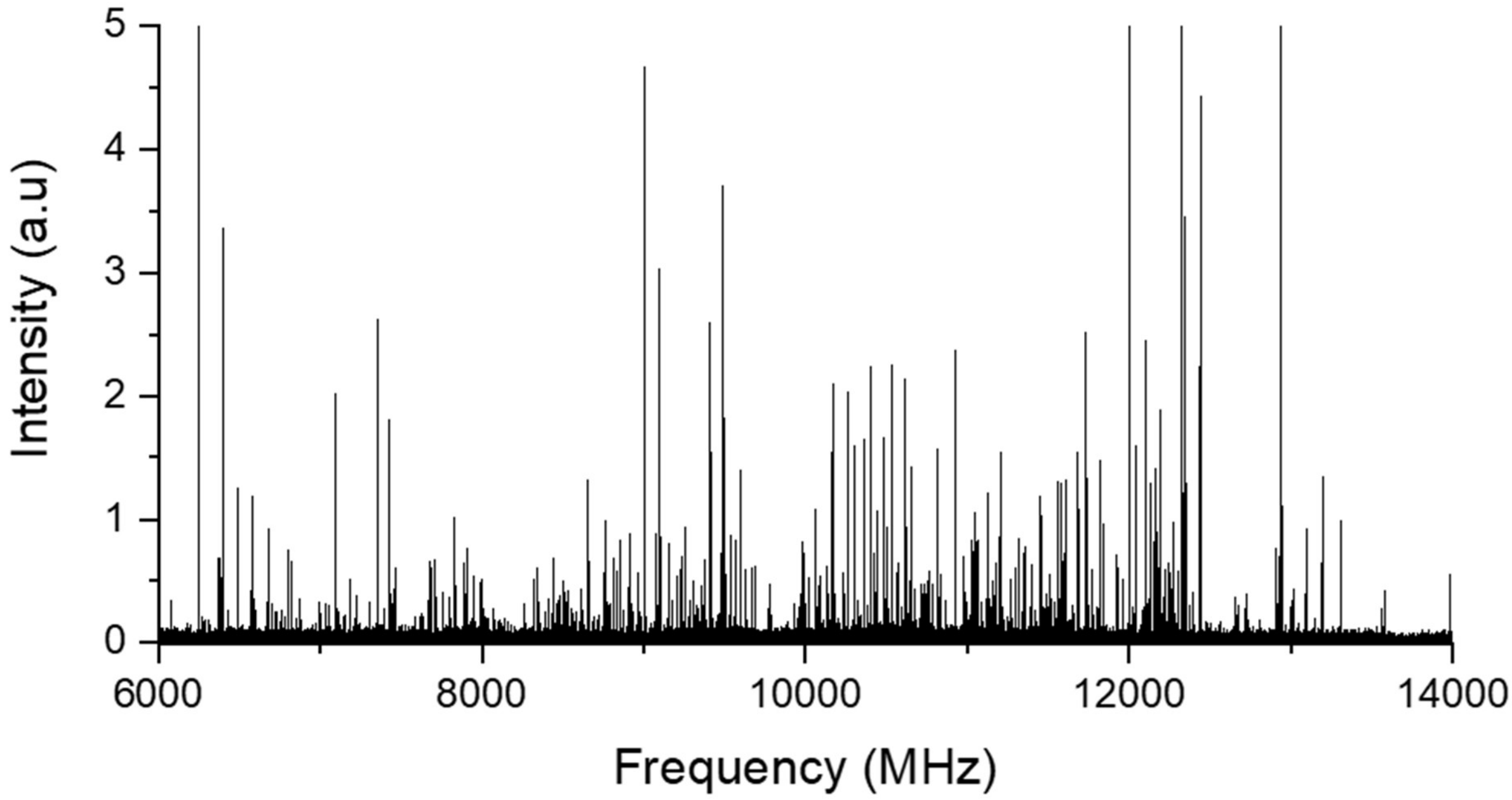

Figure S2. Relaxed potencial energy scan relaxation pathway of 7ADCA. (a) Amine inversion. (b) Amine rotation.

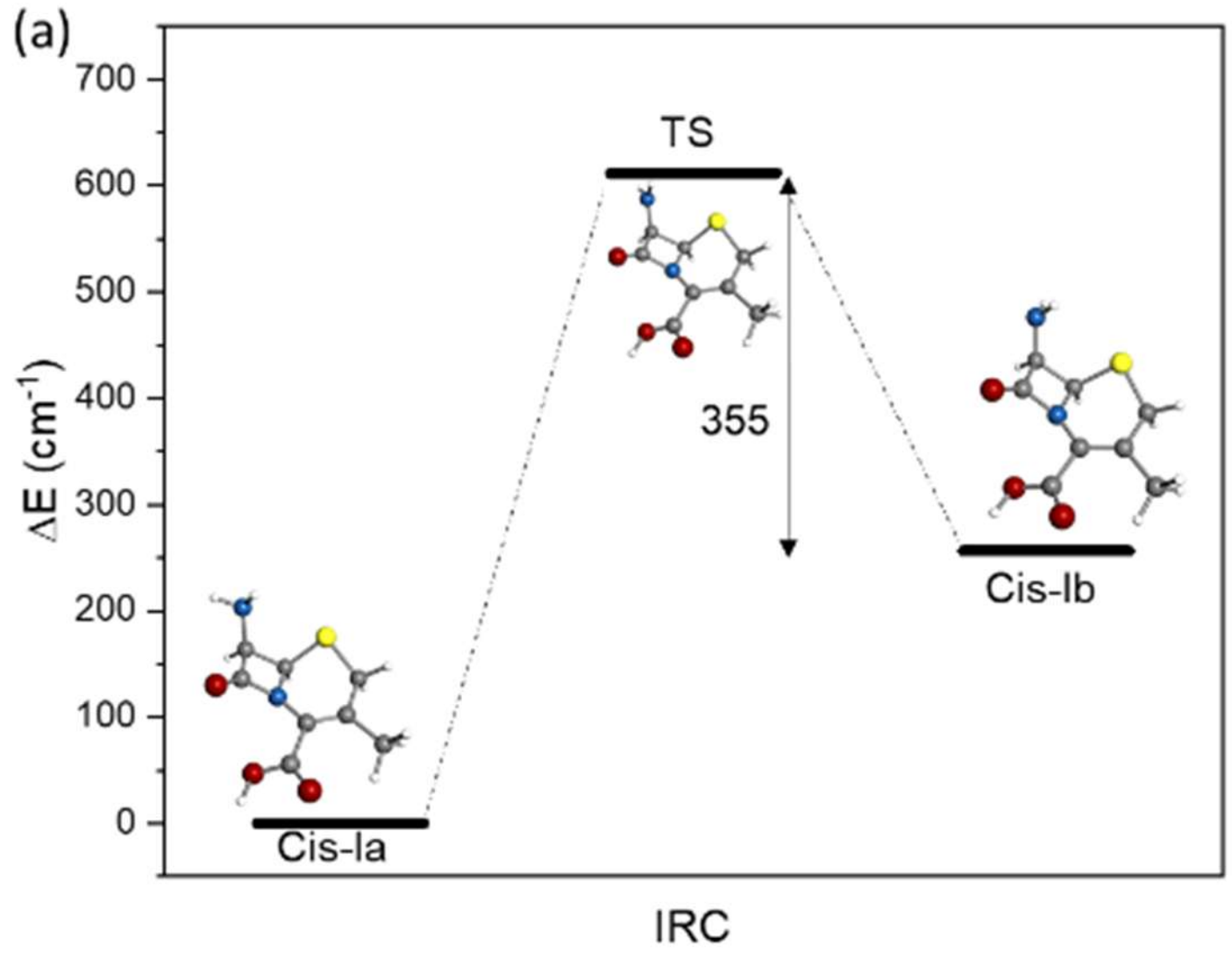

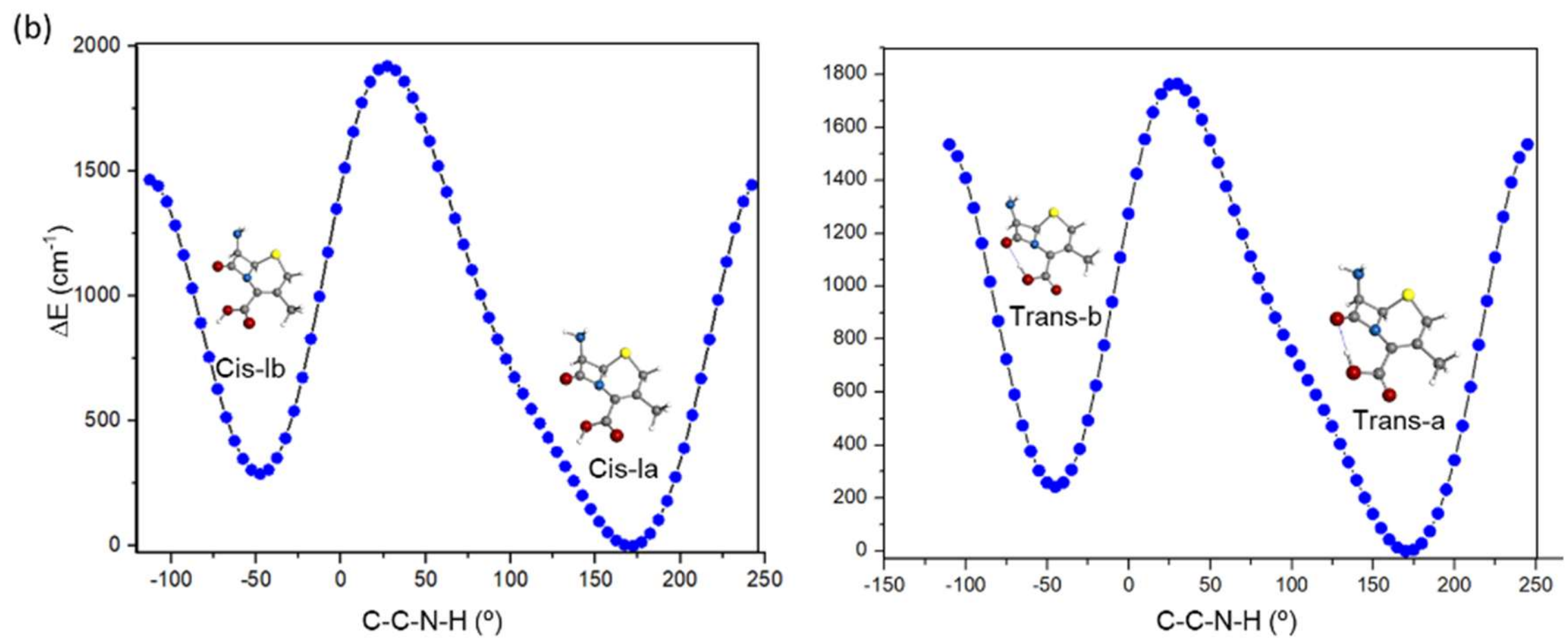

Table S5. Measured frequencies and residuals (in MHz) for rotamer I of 7-ADCA (Cis-Ia).

J´	K´$_a$	K´$_c$	I´	F´	J´´	K´´$_a$	K´´$_c$	I´´	F´´	ν_{obs}	ν_{obs}-ν_{cal}
4	3	1	4	4	3	2	2	3	3	6175.104	0.055
4	3	1	5	5	3	2	2	4	4	6175.546	-0.042
5	2	3	5	6	4	1	3	5	5	6570.024	-0.017
5	2	3	5	5	4	1	3	4	4	6570.273	-0.032
5	2	3	6	6	4	1	3	4	5	6570.596	-0.003
5	3	2	6	7	4	2	2	5	6	6822.609	-0.056
5	1	4	6	7	4	0	4	5	6	6875.910	-0.008
5	1	4	6	6	4	0	4	5	5	6876.530	0.042
5	1	4	4	4	4	0	4	3	3	6876.677	-0.019
5	2	4	5	6	4	1	4	4	5	7034.760	-0.009
5	2	4	5	5	4	1	4	4	4	7035.171	0.046
5	2	4	6	7	4	1	4	5	6	7035.491	0.009
5	2	4	6	6	4	1	4	5	5	7035.889	-0.045
8	1	7	9	10	7	2	6	8	9	7152.954	0.044
8	2	7	9	10	7	1	6	8	9	7187.789	-0.004
5	3	3	5	6	4	2	3	4	5	7219.050	0.040
5	3	3	6	7	4	2	3	5	6	7219.460	0.066
9	0	9	10	11	8	1	8	9	10	7302.449	-0.008
9	1	9	10	11	8	0	8	9	10	7302.449	-0.008
8	2	6	9	10	7	3	5	8	9	7587.667	-0.015
5	4	1	6	6	4	3	1	5	5	7668.665	0.006
5	4	2	6	7	4	3	2	5	6	7755.128	-0.018
5	4	2	5	6	4	3	2	4	5	7755.128	-0.018
6	3	3	7	8	5	2	3	6	7	7911.978	0.040
9	1	8	9	9	8	2	7	8	8	7938.751	0.034
9	2	8	9	9	8	1	7	8	8	7950.018	0.032
6	2	4	6	7	5	1	4	5	6	7994.426	-0.005
6	2	4	7	6	5	1	4	6	5	7994.798	-0.011
6	2	4	5	6	5	1	4	4	5	7995.005	0.042

6	2	4	5	5	5	1	4	4	4	7995.324	-0.028
10	0	10	11	12	9	1	9	10	11	8078.306	0.014
10	1	10	11	12	9	0	9	10	11	8078.306	0.014
6	1	5	6	7	5	0	5	5	6	8345.728	-0.028
6	1	5	6	6	5	0	5	5	5	8346.219	0.047
6	1	5	5	6	5	0	5	4	5	8346.666	0.035
6	1	5	7	7	5	0	5	6	6	8346.917	-0.006
6	2	5	6	7	5	1	5	5	6	8411.987	-0.027
6	2	5	6	6	5	1	5	5	5	8412.391	0.009
6	2	5	5	5	5	1	5	4	4	8413.312	0.052
6	2	5	7	7	5	1	5	6	6	8413.093	-0.014
6	2	5	5	6	5	1	5	4	5	8412.835	-0.027
6	3	4	6	6	5	2	4	5	5	8460.384	-0.069
6	3	4	7	8	5	2	4	6	7	8460.668	0.031
6	3	4	5	5	5	2	4	4	4	8460.918	-0.009
9	2	7	9	9	8	3	6	8	8	8499.599	-0.016
6	4	2	7	7	5	3	2	6	6	8581.053	-0.010
6	4	2	5	4	5	3	2	4	3	8581.316	0.054
10	1	9	11	12	9	2	8	10	11	8717.343	-0.002
10	2	9	10	10	9	1	8	9	9	8720.874	0.046
6	4	3	7	8	5	3	3	6	7	8833.330	-0.051
11	0	11	12	13	10	1	10	11	12	8854.148	0.001
11	1	11	12	11	10	0	10	11	10	8854.148	0.001
6	4	2	6	7	5	3	3	5	6	8976.547	0.022
6	4	2	7	8	5	3	3	6	7	8976.809	-0.021
7	3	4	7	8	6	2	4	6	7	9156.944	0.036
7	3	4	8	9	6	2	4	7	8	9157.111	-0.012
10	3	8	11	12	9	2	7	10	11	9412.214	-0.007
6	5	2	7	8	5	4	1	6	7	9470.347	-0.015
6	5	1	7	7	5	4	1	6	6	9477.639	0.010
6	5	2	7	7	5	4	2	6	6	9502.529	0.036

7	4	3	8	8	6	3	3	7	7	9503.343	0.020
7	4	3	8	9	6	3	3	7	8	9503.462	0.030
7	2	5	7	8	6	1	5	6	7	9504.983	0.039
7	2	5	7	7	6	1	5	6	6	9505.348	0.014
7	2	5	6	7	6	1	5	5	6	9505.513	0.007
7	2	5	6	6	6	1	5	5	5	9505.893	-0.013
6	5	1	7	7	5	4	2	6	6	9509.843	0.013
12	1	12	13	14	11	0	11	12	13	9630.028	0.008
12	0	12	13	14	11	1	11	12	13	9630.028	0.008
7	3	5	7	8	6	2	5	6	7	9772.284	-0.026
7	3	5	7	7	6	2	5	6	6	9772.558	0.003
7	3	5	8	9	6	2	5	7	8	9772.738	0.023
7	3	5	8	8	6	2	5	7	7	9773.014	0.038
7	4	4	7	7	6	3	4	6	6	9976.394	0.044
11	2	9	12	13	10	3	8	11	12	10129.069	0.041
11	3	9	12	13	10	2	8	11	12	10157.030	0.052
6	6	1	5	6	5	5	0	4	5	10263.581	-0.052
6	6	0	5	6	5	5	0	4	5	10263.581	-0.052
6	6	1	5	6	5	5	1	4	5	10264.265	-0.020
6	6	0	5	6	5	5	1	4	5	10264.265	-0.020
7	5	3	8	9	6	4	2	7	8	10387.368	-0.029
13	0	13	14	15	12	1	12	13	14	10405.900	-0.001
13	1	13	14	15	12	0	12	13	14	10405.900	-0.001
7	5	2	7	8	6	4	2	6	7	10428.015	0.000
7	5	3	7	8	6	4	3	6	7	10530.751	-0.028
7	5	3	8	9	6	4	3	7	8	10530.751	-0.028
8	4	4	9	9	7	3	4	8	8	10535.636	-0.003
8	3	5	9	8	7	2	5	7	8	10567.946	-0.034
8	3	5	9	10	7	2	5	8	9	10568.256	0.000
8	3	5	9	9	7	2	5	8	8	10568.561	0.053
11	3	8	12	13	10	4	7	11	12	10636.937	-0.028

8	2	6	8	9	7	1	6	7	8	11012.510	-0.011
8	2	6	9	8	7	1	6	8	7	11012.915	0.000
8	2	6	9	9	7	1	6	8	8	11013.260	-0.035
8	3	6	8	9	7	2	6	7	8	11137.197	0.010
8	3	6	8	8	7	2	6	7	7	11137.483	-0.045
8	3	6	9	10	7	2	6	8	9	11137.483	-0.045
8	3	6	9	9	7	2	6	8	8	11137.812	-0.054
14	0	14	15	16	13	1	13	14	15	11181.773	-0.015
14	1	14	15	16	13	0	13	14	15	11181.773	-0.015
8	4	5	9	10	7	3	5	8	9	11195.779	-0.010
7	6	1	8	9	6	5	1	7	8	11264.334	0.036
7	6	2	8	9	6	5	2	7	8	11270.129	0.006
7	6	1	8	9	6	5	2	7	8	11271.650	0.024
8	5	3	9	10	7	4	3	8	9	11319.251	0.032
8	5	4	9	10	7	4	4	8	9	11595.684	-0.060
9	4	5	10	11	8	3	5	9	10	11736.403	-0.003
9	4	5	10	11	8	3	5	9	10	11736.403	-0.003
8	5	3	8	9	7	4	4	7	8	11750.762	-0.016
8	5	3	9	10	7	4	4	8	9	11751.052	0.056
15	0	15	16	17	14	1	14	15	16	11957.644	-0.035
15	1	15	16	17	14	0	14	15	16	11957.643	-0.036
7	7	0	8	9	6	6	0	7	8	12038.822	-0.047
7	7	1	8	9	6	6	1	7	8	12038.822	-0.047
9	3	6	9	10	8	2	6	8	9	12095.550	-0.020
9	3	6	10	11	8	2	6	9	10	12095.879	-0.017
9	3	6	10	10	8	2	6	9	9	12096.211	0.014
9	5	4	10	10	8	4	4	9	9	12197.011	-0.007
9	5	4	10	11	8	4	4	9	10	12197.011	-0.007
8	6	2	9	10	7	5	2	8	9	12247.470	0.038
8	6	3	9	10	7	5	3	8	9	12278.215	0.007
9	5	5	10	11	8	4	5	9	10	12719.464	-0.052

16	0	16	17	16	15	1	15	16	15	12733.538	-0.034
16	1	16	17	16	15	0	15	16	15	12733.538	-0.034

Table S6. Measured frequencies and residuals (in MHz) for rotamer II of 7-ADCA (Trans-a).

J´	K´$_a$	K´$_c$	I´	F´	J´´	K´´$_a$	K´´$_c$	I´´	F´´	ν_{obs}	ν_{obs}-ν_{cal}
8	1	8	9	10	7	1	7	8	9	6383.831	0.015
6	3	3	7	8	5	3	2	6	7	6402.779	0.020
5	3	3	6	5	4	2	2	5	4	6432.218	-0.056
7	3	5	8	7	6	3	4	7	6	6783.295	0.029
8	1	7	9	10	7	1	6	8	9	7057.041	0.013
7	2	5	8	9	6	2	4	7	8	7109.538	-0.028
7	4	3	8	9	6	4	2	7	8	7302.449	-0.029
7	4	3	8	8	6	4	2	7	7	7302.449	-0.029
8	3	6	8	8	7	3	5	7	7	7627.059	0.019
5	3	2	6	6	4	2	3	5	5	7626.075	-0.027
5	3	2	4	3	4	2	3	3	4	7625.669	0.040
5	3	2	5	6	4	2	3	4	5	7624.870	-0.004
5	4	2	6	6	4	3	1	5	5	7681.783	0.035
5	4	1	6	7	4	3	2	5	6	7829.806	-0.030
8	2	6	9	10	7	2	5	8	9	7833.142	0.002
10	1	10	11	12	9	1	9	10	11	7897.696	0.009
10	0	10	11	12	9	0	9	10	11	7897.696	0.009
8	4	5	9	10	7	4	4	8	9	7989.713	0.043
8	5	4	9	9	7	5	3	8	8	8068.344	0.010
9	2	7	10	11	8	3	6	9	10	8363.839	0.017
9	3	7	10	11	8	3	6	9	10	8431.227	0.039
6	4	3	7	7	5	3	2	6	6	8469.109	-0.003
6	4	3	7	8	5	3	2	6	7	8469.507	0.029
8	3	5	9	10	7	3	4	8	9	8508.820	0.031

8	4	4	9	10	7	4	3	8	9	8521.334	-0.019
8	4	4	9	9	7	4	3	8	8	8521.532	0.008
9	2	7	10	11	8	2	6	9	10	8533.547	0.007
10	1	9	11	12	9	2	8	10	11	8555.533	0.006
10	2	9	11	12	9	2	8	10	11	8556.519	0.045
10	1	9	11	12	9	1	8	10	11	8558.698	0.038
5	5	1	6	6	4	4	0	5	5	8564.361	0.014
5	5	0	6	6	4	4	1	5	5	8568.688	0.027
11	0	11	12	13	10	0	10	11	12	8654.547	0.013
11	1	11	12	13	10	1	10	11	12	8654.547	0.013
9	4	6	10	11	8	4	5	9	10	8909.971	0.035
6	4	2	6	7	5	3	3	5	6	9000.574	0.005
6	4	2	7	8	5	3	3	6	7	9000.836	-0.047
9	6	4	10	11	8	6	3	9	10	9072.430	-0.013
9	6	4	10	10	8	6	3	9	9	9072.589	0.010
7	4	4	8	8	6	3	3	7	7	9085.756	0.001
7	4	4	8	9	6	3	3	7	8	9086.267	0.059
9	6	3	10	11	8	6	2	9	10	9105.316	-0.013
9	6	3	9	9	8	6	2	8	8	9105.568	-0.021
10	3	8	11	12	9	3	7	10	11	9209.575	0.032
10	2	8	11	12	9	2	7	10	11	9252.450	0.014
10	3	8	11	12	9	2	7	10	11	9276.955	0.044
11	2	10	12	13	10	2	9	11	12	9313.364	0.020
11	1	10	12	13	10	1	9	11	12	9314.028	0.015
9	3	6	10	10	8	3	5	9	9	9358.719	-0.055
9	3	6	10	11	8	3	5	9	10	9358.929	0.009
6	3	3	6	7	5	2	4	5	6	9367.367	0.026
6	3	3	7	8	5	2	4	6	7	9368.233	0.006
6	3	3	7	7	5	2	4	6	6	9368.938	-0.031
6	3	3	5	5	5	2	4	4	4	9369.171	0.037
9	5	4	10	11	8	5	3	9	10	9375.863	0.009

12	1	12	13	14	11	1	11	12	13	9411.406	0.011
12	0	12	13	12	11	0	11	12	11	9411.406	0.011
6	5	2	7	8	5	4	1	6	7	9534.094	-0.002
8	4	5	8	8	7	3	4	7	7	9560.853	-0.021
8	4	5	9	10	7	3	4	8	9	9561.047	-0.018
8	4	5	8	9	7	3	4	7	8	9561.371	0.011
6	5	1	7	8	5	4	2	6	7	9572.001	0.011
10	4	7	11	12	9	4	6	10	11	9777.011	0.020
11	2	9	11	11	10	3	8	10	10	9965.846	0.045
9	4	6	10	10	8	3	5	9	9	9961.792	-0.001
11	3	9	12	13	10	3	8	11	12	9974.153	0.039
11	2	9	12	13	10	2	8	11	12	9990.255	0.019
11	3	9	12	13	10	2	8	11	12	9998.629	0.040
10	7	3	11	12	9	7	2	10	11	10068.372	0.024
10	3	7	11	11	9	3	6	10	10	10085.447	-0.037
10	3	7	11	12	9	3	6	10	11	10085.655	0.001
10	3	7	10	11	9	3	6	9	10	10085.870	0.041
10	5	6	11	12	9	5	5	10	11	10092.553	0.011
10	6	5	11	12	9	6	4	10	11	10131.152	0.032
13	1	13	14	15	12	1	12	13	14	10168.272	0.004
13	0	13	14	15	12	0	12	13	14	10168.272	0.004
10	6	4	11	11	9	6	3	10	10	10237.090	-0.018
6	6	1	7	7	5	5	0	6	6	10360.001	0.003
6	6	0	7	7	5	5	1	6	6	10360.767	0.000
7	5	3	8	8	6	4	2	7	7	10438.697	-0.045
7	5	3	8	9	6	4	2	7	8	10438.928	-0.009
11	4	8	12	13	10	4	7	11	12	10597.743	-0.024
7	5	2	8	9	6	4	3	7	8	10616.329	-0.041
10	5	5	11	12	9	5	4	10	11	10627.633	-0.046
10	5	5	11	11	9	5	4	10	10	10627.873	0.030
10	4	6	11	12	9	4	5	10	11	10726.865	-0.025

12	3	10	13	14	11	3	9	12	13	10732.702	0.039
12	2	10	13	14	11	2	9	12	13	10738.332	0.035
12	3	10	13	14	11	2	9	12	13	10741.064	0.048
11	3	8	12	13	10	3	7	11	12	10766.277	0.009
14	1	14	15	16	13	1	13	14	15	10925.154	0.006
14	0	14	15	16	13	0	13	14	15	10925.154	0.006
11	5	7	12	13	10	5	6	11	12	11038.462	0.006
11	7	5	12	13	10	7	4	11	12	11126.243	0.029
11	7	4	12	13	10	7	3	11	12	11158.551	-0.021
11	6	6	12	13	10	6	5	11	12	11179.257	-0.048
8	5	4	9	9	7	4	3	8	8	11204.463	-0.007
8	5	4	9	10	7	4	3	8	9	11204.757	-0.021
7	6	2	7	7	6	5	1	6	6	11344.706	-0.021
7	6	2	8	9	6	5	1	7	8	11344.895	0.036
7	6	1	8	9	6	5	2	7	8	11353.193	0.038
12	4	9	12	12	11	4	8	11	11	11385.588	0.016
11	6	5	12	13	10	6	4	11	12	11445.826	-0.043
11	6	5	12	12	10	6	4	11	11	11446.050	0.010
12	3	9	13	14	11	3	8	12	13	11462.527	-0.003
12	4	8	13	14	11	5	7	12	13	11465.707	-0.014
13	3	11	14	15	12	3	10	13	14	11489.094	0.004
13	2	11	14	15	12	2	10	13	14	11490.943	-0.013
12	4	9	13	14	11	3	8	12	13	11511.662	0.003
14	2	13	15	16	13	2	12	14	15	11583.178	-0.012
14	1	13	15	14	13	1	12	14	13	11583.178	-0.012
11	4	7	12	13	10	4	6	11	12	11612.500	-0.007
15	1	15	16	17	14	1	14	15	16	11682.022	-0.009
15	0	15	16	17	14	0	14	15	16	11682.022	-0.009
8	5	3	8	9	7	4	4	7	8	11774.146	-0.030
8	5	3	9	10	7	4	4	8	9	11774.434	0.039
9	5	5	10	10	8	4	4	9	9	11780.144	-0.043

9	5	5	9	9	8	4	4	8	8	11780.413	0.035
9	5	5	9	10	8	4	4	8	9	11780.739	-0.054
11	5	6	12	13	10	5	5	11	12	11843.188	-0.019
12	5	8	13	14	11	5	7	12	13	11928.361	-0.006
12	8	5	13	14	11	8	4	12	13	12107.658	0.000
12	8	4	13	14	11	8	3	12	13	12116.299	0.008
13	3	10	14	15	12	4	9	13	14	12136.127	0.007
13	4	10	13	14	12	4	9	12	13	12154.099	-0.008
13	3	10	14	15	12	3	9	13	14	12185.249	-0.001
12	7	6	12	13	11	7	5	11	12	12198.857	-0.009
13	4	10	14	15	12	3	9	13	14	12203.160	0.022
14	2	12	15	16	13	3	11	14	15	12244.661	-0.073
14	3	12	15	16	13	3	11	14	15	12244.936	-0.058
14	2	12	15	16	13	2	11	14	15	12245.548	-0.039
14	3	12	15	16	13	2	11	14	15	12245.848	0.002
12	7	5	13	14	11	7	4	12	13	12298.287	0.020
8	6	3	8	9	7	5	2	7	8	12306.169	0.013
15	2	14	16	17	14	2	13	15	16	12339.865	-0.015
15	1	14	16	17	14	1	13	15	16	12339.865	-0.015
12	4	8	13	14	11	4	7	12	13	12352.095	-0.010
8	6	2	9	10	7	5	3	8	9	12353.894	0.027
16	0	16	17	18	15	0	15	16	17	12438.897	-0.022
12	6	6	13	14	11	6	5	12	13	12722.028	0.012
14	4	11	15	16	13	4	10	14	15	12913.223	-0.027
14	3	11	15	16	13	3	10	14	15	12924.943	-0.005
12	5	7	13	14	11	5	6	12	13	12936.713	-0.031
15	3	13	16	17	14	3	12	15	16	13000.976	-0.032
15	2	13	16	17	14	2	12	15	16	13000.976	-0.032
13	4	9	14	15	12	4	8	13	14	13017.713	-0.021
16	2	15	17	18	15	2	14	16	17	13096.590	-0.026
16	1	15	17	18	15	1	14	16	17	13096.590	-0.026

8	7	1	9	9	7	6	2	8	8	13144.545	-0.015
17	0	17	18	19	16	0	16	17	18	13195.771	-0.037
17	1	17	18	19	16	1	16	17	18	13195.771	-0.037

Table S7. Topological properties at the bond critical points (BCP) for 7-ADCA Cis-Ia conformer calculated by QTAIM analysis at MP2 method.

Bond	ρ^a	$\nabla^2\rho^b$	V^c	G^d	V/G	H^e	ε^f
C2 C1	0.3229	-0.8879	-0.4967	0.1373	3.6163	-0.3593	0.3893
C3 C2	0.2497	-0.5877	-0.2831	0.0681	4.1582	-0.2150	0.0408
S4 C3	0.1773	-0.2736	-0.1756	0.0536	3.2762	-0.1220	0.0906
N6 C1	0.2849	-0.7543	-0.5794	0.1954	2.9651	-0.3840	0.0878
S4 C5	0.1816	-0.2854	-0.1846	0.0566	3.2602	-0.1280	0.0904
C5 N6	0.2617	-0.6694	-0.4440	0.1384	3.2095	-0.3057	0.0150
N6 C7	0.2894	-0.7817	-0.5543	0.1794	3.0891	-0.3749	0.0955
C8 C7	0.2436	-0.5480	-0.2703	0.0666	4.0555	-0.2036	0.0739
C5 C8	0.2342	-0.5054	-0.2535	0.0636	3.9875	-0.1899	0.0360
C7 O9	0.4064	0.0864	-1.4046	0.7131	1.9697	-0.6915	0.0768
C10 H21	0.2769	-0.9419	-0.3264	0.0455	7.1807	-0.2809	0.0090
C2 C10	0.2498	-0.5968	-0.2880	0.0694	4.1496	-0.2186	0.0425
N11 C8	0.2764	-0.7776	-0.4446	0.1251	3.5542	-0.3195	0.0464
C1 C12	0.2668	-0.6922	-0.3098	0.0684	4.5311	-0.2414	0.1341
H21 O13	0.0142	0.0526	-0.0096	0.0114	0.8438	0.0018	0.1673
C12 O13	0.4053	-0.0498	-1.3741	0.6808	2.0183	-0.6933	0.1093
C12 O14	0.2952	-0.4169	-0.7717	0.3337	2.3123	-0.4380	0.0068
H15 C5	0.2796	-0.9572	-0.3226	0.0416	7.7458	-0.2809	0.0228
H16 C3	0.2734	-0.9157	-0.3202	0.0456	7.0170	-0.2746	0.0080
H17 C3	0.2714	-0.9004	-0.3164	0.0457	6.9275	-0.2708	0.0130
H18 C8	0.2776	-0.9480	-0.3231	0.0431	7.5025	-0.2801	0.0158
H19 C10	0.2701	-0.8967	-0.3206	0.0482	6.6499	-0.2724	0.0111
C10 H20	0.2689	-0.8897	-0.3169	0.0472	6.7109	-0.2696	0.0100
N11 H22	0.3334	-1.5648	-0.5249	0.0668	7.8534	-0.4580	0.0488
N11 H23	0.3339	-1.5798	-0.5265	0.0658	8.0057	-0.4607	0.0456
O14 H24	0.3531	-2.4877	-0.7696	0.0738	10.4230	-0.6958	0.0170

[a] Electron density at the BCP. [b] Laplacian at the BCP (au). [c] Potential energy density (au). [d] Gradient kinetic energy density (au). [e] Total electronic energy density (au). [f] Bond ellipticity.

Table S8. Topological properties at the bond critical points (BCP) for 7-ADCA Trans-a conformer calculated by QTAIM analysis at MP2 method.

Bond	ρ^a	$\nabla^2\rho^b$	V^c	G^d	V/G	H^e	ε^f
C2 C1	0.3225	-0.8825	-0.4966	0.1380	3.5989	-0.3586	0.3978
C3 C2	0.2490	-0.5843	-0.2815	0.0677	4.1581	-0.2138	0.0383
S4 C3	0.1774	-0.2740	-0.1753	0.0534	3.2820	-0.1219	0.0897
N6 C1	0.2738	-0.6921	-0.5563	0.1916	2.9030	-0.3646	0.0843
C5 N6	0.2568	-0.6429	-0.4342	0.1367	3.1756	-0.2975	0.0194
S4 C5	0.1837	-0.2969	-0.1898	0.0578	3.2846	-0.1320	0.0955
C8 C7	0.2471	-0.5672	-0.2783	0.0683	4.0770	-0.2101	0.0747
N6 C7	0.2988	-0.8146	-0.6049	0.2006	3.0150	-0.4043	0.1052
C5 C8	0.2342	-0.5055	-0.2534	0.0635	3.9903	-0.1899	0.0351
C7 O9	0.3961	0.0057	-1.3359	0.6687	1.9979	-0.6673	0.0698
C10 H19	0.2789	-0.9558	-0.3283	0.0446	7.3521	-0.2836	0.0094
C2 C10	0.2493	-0.5949	-0.2881	0.0697	4.1340	-0.2184	0.0402
N11 C8	0.2779	-0.7841	-0.4463	0.1251	3.5664	-0.3212	0.0461
C1 C12	0.2560	-0.6297	-0.2884	0.0655	4.4033	-0.2229	0.1012
C12 O13	0.4075	-0.0608	-1.3828	0.6838	2.0222	-0.6990	0.1285
O9 H24	0.0400	0.1257	-0.0368	0.0341	1.0793	-0.0027	0.0205
H19 O13	0.0163	0.0612	-0.0112	0.0133	0.8453	0.0021	0.1267
C12 O14	0.3018	-0.4012	-0.8037	0.3517	2.2852	-0.4520	0.0208
H15 C5	0.2802	-0.9622	-0.3226	0.0410	7.8604	-0.2816	0.0227
H16 C3	0.2747	-0.9231	-0.3219	0.0455	7.0666	-0.2763	0.0085
H17 C3	0.2717	-0.9022	-0.3167	0.0456	6.9494	-0.2711	0.0126
H18 C8	0.2781	-0.9520	-0.3232	0.0426	7.5869	-0.2806	0.0157
H20 C10	0.2683	-0.8859	-0.3169	0.0477	6.6408	-0.2692	0.0120
C10 H21	0.2703	-0.8974	-0.3210	0.0483	6.6449	-0.2727	0.0122
N11 H22	0.3334	-1.5742	-0.5262	0.0663	7.9315	-0.4599	0.0492
N11 H23	0.3340	-1.5882	-0.5280	0.0655	8.0652	-0.4625	0.0462

[a] Electron density at the BCP. [b] Laplacian at the BCP (au). [c] Potential energy density (au). [d] Gradient kinetic energy density (au). [e] Total electronic energy density (au). [f] Bond ellipticity.

Table S9. Nuclear charge calculated using the QTAIM analysis for conformers Cis-Ia and Trans-a.

Atom	Cis-Ia	Trans-a
**C1**	0.36	0.31
**C2**	0.03	0.03
**C3**	-0.10	-0.10
**S4**	0.06	0.07
**C5**	0.23	0.21
**N6**	-1.14	-1.17
**C7**	1.39	1.38
**C8**	0.39	0.40
**O9**	-1.13	-1.14
**C10**	0.01	0.01
**N11**	-1.05	-1.05
**C12**	1.57	1.62
**O13**	-1.13	-1.12
**O14**	-1.12	-1.17
**H15**	0.05	0.06
**H16**	0.05	0.05
**H17**	0.04	0.05
**H18**	0.05	0.06
**H19**	0.01	0.07
**H20**	0.03	0.03
**H21**	0.05	0.01
**H22**	0.37	0.37
**H23**	0.37	0.37
**H24**	0.60	0.64

Figure S3. Cephalosporin ligands collected from several PDB.

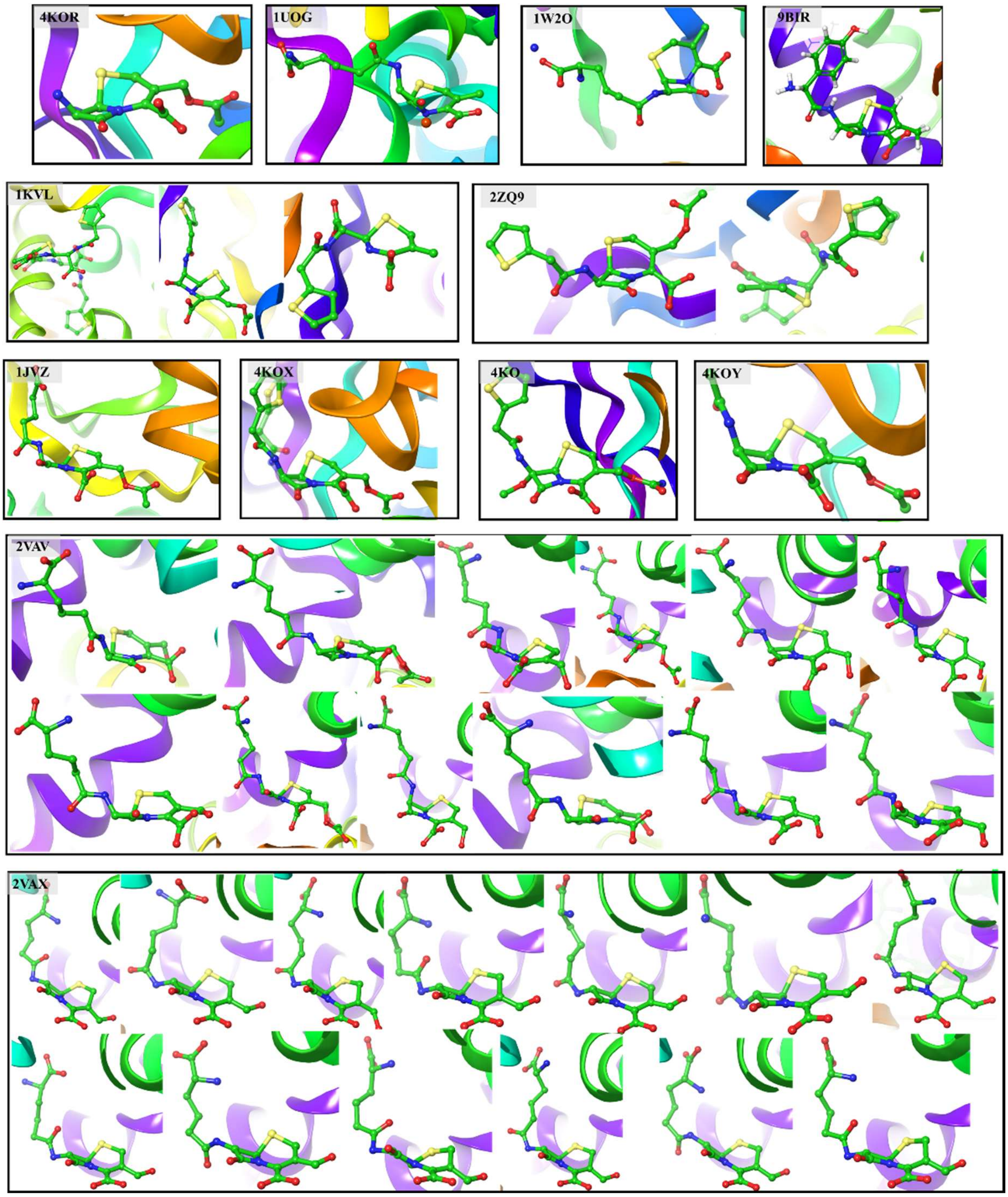

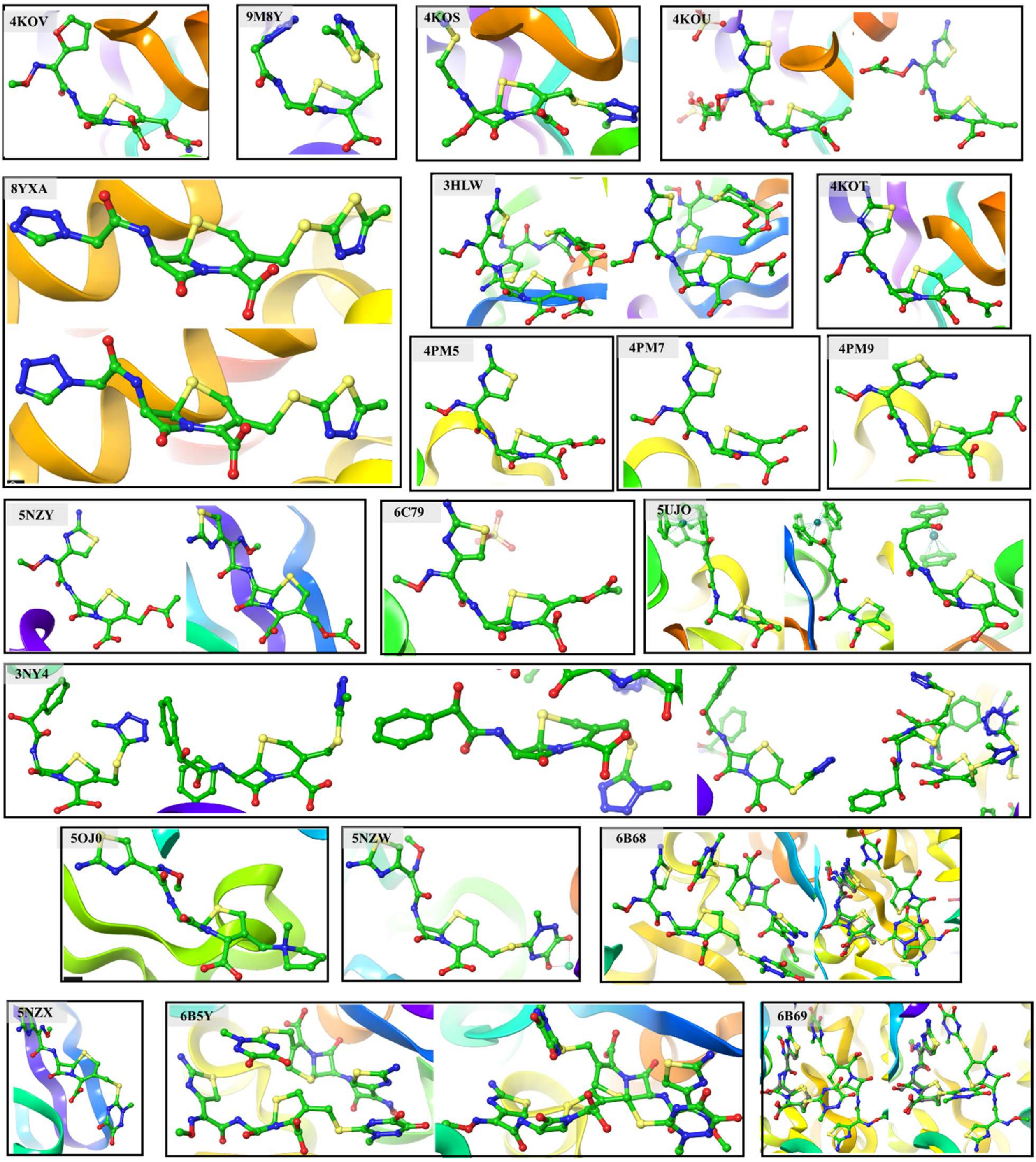
4KOV
9M8Y
4KOS
4KOU
8YXA
3HLW
4KOT
4PM5
4PM7
4PM9
5NZY
6C79
5UJO
3NY4
5OJ0
5NZW
6B68
5NZX
6B5Y
6B69

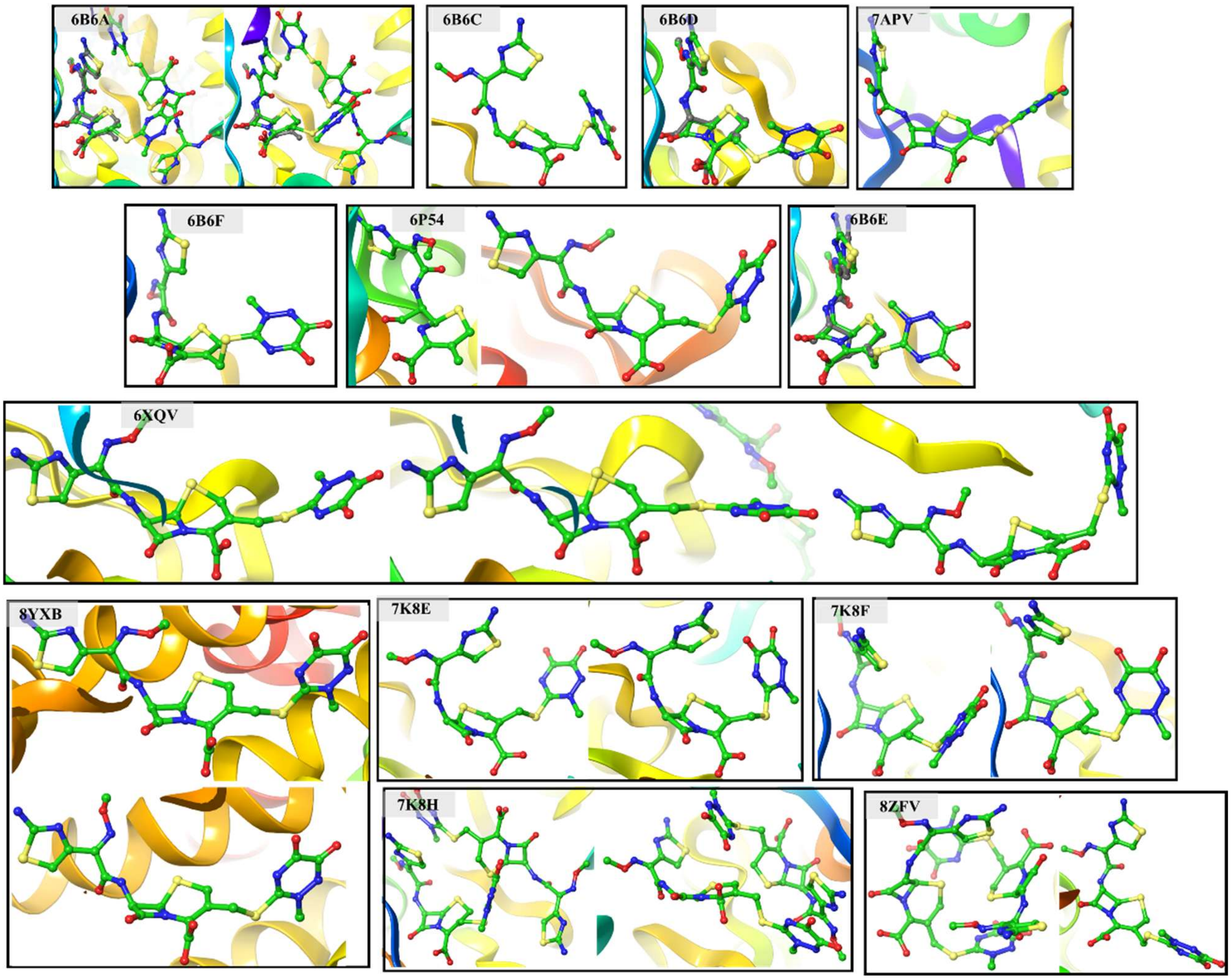

6B6A
6B6C
6B6D
7APV
6B6F
6P54
6B6E
6XQV
8YXB
7K8E
7K8F
7K8H
8ZFV

Table S10. Structural hallmarks ($d_{O \cdots C}$ distances and $\alpha_{O \cdots C=O}$ angles) of the $n_O \rightarrow \pi^*_{C=O}$ interaction for the 111 cephalosporin-ligand complexes retrieved from the Protein Data Bank (PDB).

Protein	O•••C bond distance (Å)	O•••C=O Bürgi-Dunitz-angle (°)
**1JVZ**	3.487	109
**1KVL_1**	4.768	118
**1KVL_2**	3.316	86
**1KVL_3**	7.105	105
**1UOGA**	2.884	138
**1W2O**	3.390	135
**2VAV_1**	3.113	123
**2VAV_2**	3.158	125
**2VAV_3**	2.994	121
**2VAV_4**	3.201	125
**2VAV_5**	3.150	91
**2VAV_6**	2.953	108
**2VAV_7**	2.978	114
**2VAV_8**	3.032	118
**2VAV_9**	3.235	115
**2VAV_10**	3.139	91
**2VAV_11**	3.270	130
**2VAX_1**	3.121	152
**2VAX_2**	3.173	154
**2VAX_3**	3.173	152
**2VAX_4**	3.129	154
**2VAX_5**	3.376	160
**2VAX_6**	3.226	154
**2VAX_7**	3.178	151
**2VAX_8**	3.103	151
**2VAX_9**	3.363	157
**2VAX_10**	3.140	155
**2VAX_11**	3.101	153
**2VAX_12**	3.108	155
**2ZQ9_1**	3.304	122
**2ZQ9_2**	4.244	103
**3HLW_1**	3.197	102
**3HLW_2**	3.081	127
**3HLW_3**	3.228	101
**3HLW_4**	3.059	123
**3NY4_1**	3.773	149
**3NY4_2**	3.626	132
**3NY4_3**	3.304	132

Protein	O•••C bond distance (Å)	O•••C=O Bürgi-Dunitz-angle (°)
**3NY4_4**	3.175	144
**3NY4_5**	3.273	112
**4KOR**	3.215	127
**4KOS**	3.266	127
**4KOT**	3.239	126
**4KOU_1**	3.160	123
**4KOU_2**	3.221	119
**4KOV**	3.278	123
**4KOW**	3.164	124
**4KOX**	3.198	120
**4KOY**	3.244	126
**4PM5**	3.604	107
**4PM7**	3.222	113
**4PM9**	3.875	106
**5NZW**	3.322	144
**5NZX**	3.174	113
**5NZY_1**	3.136	135
**5NZY_2**	3.181	126
**5OJ0**	3.758	153
**5UJ0_1**	4.492	122
**5UJ0_2**	4.467	121
**5UJ0_3**	3.126	131
**6B5Y_1**	3.393	104
**6B5Y_2**	3.422	112
**6B5Y_3**	3.176	117
**6B5Y_4**	3.087	90
**6B6A_1**	3.326	92
**6B6A_2**	3.186	91
**6B6A_3**	3.802	113
**6B6A_4**	3.113	110
**6B6A_5**	3.163	89
**6B6A_6**	4.031	100
**6B6C**	2.941	90
**6B6D_1**	3.919	85
**6B6D_2**	3.259	127
**6B6E_1**	3.030	106
**6B6E_2**	3.488	86
**6B6F**	3.047	98
**6B68_1**	3.337	112

Protein	O•••C bond distance (Å)	O•••C=O Bürgi-Dunitz-angle (°)
**6B68_2**	3.235	95
**6B68_3**	4.035	107
**6B68_4**	3.198	97
**6B68_5**	3.240	93
**6B69_1**	3.130	104
**6B69_2**	3.042	85
**6B69_3**	3.736	91
**6B69_4**	3.131	100
**6B69_5**	3.008	89
**6B69_6**	3.346	98
**6C79**	3.118	94
**6P54_1**	4.229	103
**6P54_2**	3.290	131
**6XQV_1**	3.366	117
**6XQV_2**	3.448	116
**6XQV_3**	3.381	155
**7APV**	3.847	119
**7K8E_1**	3.273	94
**7K8E_2**	3.358	122
**7K8F_1**	3.327	98
**7K8F_2**	3.287	115
**7K8H_1**	3.165	113
**7K8H_2**	3.305	91
**7K8H_3**	3.081	107
**7K8H_4**	3.240	97
**8YXA_1**	3.161	120
**8YXA_2**	3.170	102
**8YXB_1**	3.291	96
**8YXB_2**	3.330	96
**8ZFV_1**	3.385	163
**8ZFV_2**	3.195	132
**8ZFV_3**	3.055	120
**9BIR**	3.151	126
**9M8Y**	3.696	166
**PDB average**	3.360±0.489	118±21
**Cis-Ia**	3.248	123
**Trans-a**	3.388	168

Table S11. Theoretical spectroscopic parameters of 7-ADCA at B3LYP-D3(BJ)/6-311++(d,p) using the IEFPCM model.

Parameters	Cis-Ib	Cis-Ia	Cis-IIb	Cis-IIa	Trans-b	Trans-a
A[a]	873	875	880	882	884	886
B	590	590	584	583	588	588
C	383	383	382	382	375	375
$\lvert\mu_a\rvert$[b]	1.1	0.5	-1.3	-1.9	5.8	5.4
$\lvert\mu_b\rvert$	6.8	3.7	9.4	6.3	8.1	4.9
$\lvert\mu_c\rvert$	5.4	4.1	2.5	1.4	2.5	1.4
$\chi_{aa(r)}$[c]	1.97	1.98	1.99	1.99	1.90	1.91
$\chi_{bb(r)}$	0.77	0.80	0.83	0.85	0.86	0.89
$\chi_{cc(r)}$	-2.74	-2.77	-2.81	-2.84	-2.76	-2.80
$\chi_{aa(a)}$	2.41	0.42	2.37	0.43	2.42	0.74
$\chi_{bb(a)}$	-4.10	-1.76	-4.14	-1.88	-4.26	-2.12
$\chi_{cc(a)}$	1.69	1.33	1.76	1.44	1.84	1.38
ΔE[d]	0	75	137	209	253	315
ΔE_{ZPE}	0	64	119	176	221	269
ΔG	0	62	75	125	28	174

[a]A, B, and C represent the rotational constants (in MHz); [b]μ_a, μ_b and μ_c are the electric dipole moment components in Debyes (observed or not observed for experimental values). [c]χ_{aa}, χ_{bb}, and χ_{cc}, are the diagonal elements of the ^{14}N nuclear quadrupole coupling tensor (in MHz); (r) and (a) correspond to the β-lactam ring and amine ^{14}N nuclei, respectively. Standard error in parentheses expressed in units of the last digit. [d]ΔE, ΔE_{ZPE}, ΔG are the electronic energy difference, the energy difference including zero-point vibrational corrections, and the Gibbs free energy difference (1 atm, 298 K), respectively.

Table S12. Theoretical spectroscopic parameters for the most stable conformers of 7-ADCA-water complex at B3PLYP-D3(BJ)/6-311++(d,p).

Parameters	1w-Cis-Ia-1	1w-Cis-I-2	1w-Cis-Ib-1	1w-Cis-Ib-2	1w-Cis-IIa-1
A[a]	783	677	777	673	794
B	402	492	404	494	395
C	284	309	284	309	283
$\lvert\mu_a\rvert$[b]	0.8	3.1	1.9	4.6	0.4
$\lvert\mu_b\rvert$	1.3	0.9	2.9	1.9	1.6
$\lvert\mu_c\rvert$	0.6	0.4	1.3	0.9	0.4
$\chi_{aa(r)}$[c]	1.46	1.32	1.46	1.29	1.58
$\chi_{bb(r)}$	1.46	1.87	1.45	1.87	1.44
$\chi_{cc(r)}$	-2.92	-3.18	-2.91	-3.16	-3.02
$\chi_{aa(a)}$	-2.10	-3.94	2.47	0.58	-2.00
$\chi_{bb(a)}$	0.86	2.12	-4.50	-2.89	0.63
$\chi_{cc(a)}$	1.25	1.82	2.03	2.31	1.37
ΔE[d]	0	120	277	365	325
ΔE_{ZPE}	0	81	246	290	324
ΔG	0	152	264	373	290

[a]A, B, and C represent the rotational constants (in MHz); [b]μ_a, μ_b and μ_c are the electric dipole moment components in Debyes (observed or not observed for experimental values). [c]χ_{aa}, χ_{bb}, and χ_{cc}, are the diagonal elements of the ^{14}N nuclear quadrupole coupling tensor (in MHz); (r) and (a) correspond to the β-lactam ring and amine ^{14}N nuclei, respectively. Standard error in parentheses expressed in units of the last digit. [d]ΔE, ΔE_{ZPE}, ΔG are the electronic energy difference, the energy difference including zero-point vibrational corrections, and the Gibbs free energy difference (1 atm, 298 K), respectively.

Table S13. Theoretical spectroscopic parameters for the most stable conformers of 7-ADCA-2water complex at B3PLYP-D3(BJ)/6-311++(d,p).

Parameters	2W-Cis-Ia-1	2W-Cis-Ia-2	2W-Cis-Ib-1	2W-Cis-IIa-1	2W-Cis-IIa-2
A[a]	614	640	611	646	638
B	356	354	357	347	349
C	246	257	246	244	245
$\|\mu_a\|$[b]	3.4	2.9	5.0	2.3	2.8
$\|\mu_b\|$	0.5	-2.2	-0.5	-2.2	-0.8
$\|\mu_c\|$	-0.3	-1.0	-0.9	-1.2	0.0
$\chi_{aa(r)}$[c]	1.28	1.32	1.25	1.60	1.65
$\chi_{bb(r)}$	1.95	1.91	1.96	1.84	1.85
$\chi_{cc(r)}$	-3.22	-3.23	-3.21	-3.44	-3.50
$\chi_{aa(a)}$	-3.99	-3.91	0.18	-4.02	-4.00
$\chi_{bb(a)}$	2.27	2.16	-2.55	1.94	1.88
$\chi_{cc(a)}$	1.72	1.75	2.37	2.08	2.12
ΔE[d]	0	84	197	221	339
ΔE_{ZPE}	0	159	165	238	305
ΔG	0	354	173	393	299

[a]A, B, and C represent the rotational constants (in MHz); [b]μ_a, μ_b and μ_c are the electric dipole moment components in Debyes (observed or not observed for experimental values). [c]χ_{aa}, χ_{bb}, and χ_{cc}, are the diagonal elements of the ^{14}N nuclear quadrupole coupling tensor (in MHz); (r) and (a) correspond to the β-lactam ring and amine ^{14}N nuclei, respectively. Standard error in parentheses expressed in units of the last digit. [d]ΔE, ΔE_{ZPE}, ΔG are the electronic energy difference, the energy difference including zero-point vibrational corrections, and the Gibbs free energy difference (1 atm, 298 K), respectively.

Table S14. Theoretical spectroscopic parameters for the most stable conformers of 7-ADCA-3water complex at B3PLYP-D3(BJ)/6-311++(d,p).

Parameters	3W-Cis-Ia-1	3W-Cis-Ib-1	3W-Cis-IIa-1	3W-Cis-Ia-2	3W-Cis-Iia-2
A[a]	561	557	575	574	576
B	273	274	258	264	259
C	198	198	190	208	192
$\|\mu_a\|$[b]	-4.0	-5.4	-2.8	-3.1	-2.0
$\|\mu_b\|$	-0.1	-1.3	-1.9	-0.8	-1.3
$\|\mu_c\|$	-1.1	-0.5	0.1	1.3	1.5
$\chi_{aa(r)}$[c]	1.42	1.41	1.53	1.32	1.57
$\chi_{bb(r)}$	1.82	1.82	1.90	1.96	1.91
$\chi_{cc(r)}$	-3.24	-3.24	-3.43	-3.29	-3.49
$\chi_{aa(a)}$	-3.58	1.17	-4.07	-4.12	-4.04
$\chi_{bb(a)}$	1.79	-3.52	2.05	2.36	2.00
$\chi_{cc(a)}$	1.79	2.35	2.01	1.75	2.05
ΔE[d]	0	196	139	107	129
ΔE_{ZPE}	0	164	168	181	203
ΔG	0	179	91	158	177

[a]A, B, and C represent the rotational constants (in MHz); [b]μ_a, μ_b and μ_c are the electric dipole moment components in Debyes (observed or not observed for experimental values). [c]χ_{aa}, χ_{bb}, and χ_{cc}, are the diagonal elements of the ^{14}N nuclear quadrupole coupling tensor (in MHz); (r) and (a) correspond to the β-lactam ring and amine ^{14}N nuclei, respectively. Standard error in parentheses expressed in units of the last digit. [d]ΔE, ΔE_{ZPE}, ΔG are the electronic energy difference, the energy difference including zero-point vibrational corrections, and the Gibbs free energy difference (1 atm, 298 K), respectively.

Figure S4. (a) $n \rightarrow \pi^*$intramolecular interaction of the detected species of 7-ADCA and its water clusters up to three water molecules. The E(2) value obtained by the NBO analysis is given in kcal.mol^{-1}; (b) NCI-plot analysis of the most stable conformers of 7-ADCA. (b) Five most stable conformers of 7-ADCA and its water clusters up to three water molecules. The relative energies ($\Delta E_{ZPE}/\Delta G$) in cm^{-1} at B3LYP-D3(BJ)/6-311++G(d,p) is also given.

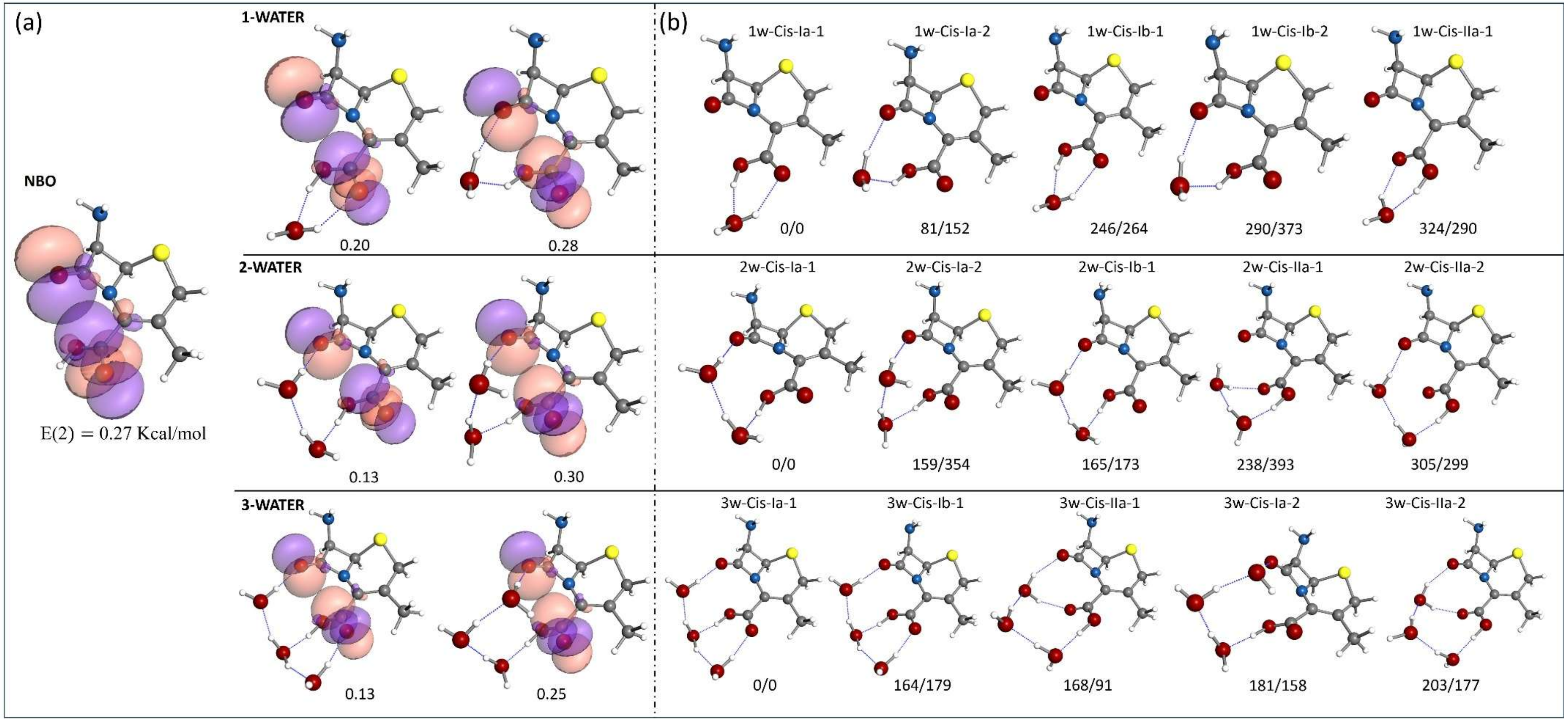

Supplement: Supplementary file 1 [file jz6c01886_si_001.pdf]
